# Barriers and facilitators to healthcare professional behaviour change in clinical trials using the Theoretical Domains Framework: a case study of a trial of individualized temperature-reduced haemodialysis

**DOI:** 10.1186/s13063-017-1965-9

**Published:** 2017-05-22

**Authors:** Justin Presseau, Brittany Mutsaers, Ahmed A. Al-Jaishi, Janet Squires, Christopher W. McIntyre, Amit X. Garg, Manish M. Sood, Jeremy M. Grimshaw, on behalf of the Major outcomes with personalized dialysate TEMPerature (MyTEMP) investigators, Amit Bagga, Andre Charest, Andrew Steele, Arsh Jain, Charmaine Lok, David Berry, David Perkins, Rebecca Harvey, Davinder Wadehra, Derek Benjamin, Eduard Iliescu, Eli Rabin, Garth Hanson, Gihad Nesrallah, Joanna Sasal, Jessica M. Sontrop, Laura Gregor, Leo Lam, Malvinder Parmar, Matthew Oliver, Michael Walsh, Nicole Delbrouck, Patricia Chan, Paul Tam, Paul Watson, Peter Blake, Phillip Zager, PJ Devereaux, Reem Mustafa, Rey Acedillo, Richard Goluch, Ron Wald, Sanjay Pandeya, Stephanie Dixon, Tanya Schulman, Walter Wodchis

**Affiliations:** 1 Clinical Epidemiology Program, The Ottawa Hospital Research Institute, General Campus, 501 Smyth Road, Ottawa, Canada; 2 School of Epidemiology, Public Health and Preventive Medicine, University of Ottawa, Ottawa, Canada; 3 School of Psychology, University of Ottawa, Ottawa, Canada; 4 Department of Health Research Methods, Evidence, and Impact, McMaster University, Hamilton, ON Canada; 5 School of Nursing, University of Ottawa, Ottawa, Canada; 6 Division of Nephrology, Departments of Medicine, Epidemiology and Biostatistics, Western University, London, ON Canada; 7 Department of Medicine, University of Ottawa, Ottawa, Canada

**Keywords:** Dialysate temperature, Haemodialysis, Theoretical Domains Framework, Trial implementation

## Abstract

**Background:**

Implementing the treatment arm of a clinical trial often requires changes to healthcare practices. Barriers to such changes may undermine the delivery of the treatment making it more likely that the trial will demonstrate no treatment effect. The ‘Major outcomes with personalized dialysate temperature’ (MyTEMP) is a cluster-randomised trial to be conducted in 84 haemodialysis centres across Ontario, Canada to investigate whether there is a difference in major outcomes with an individualized dialysis temperature (IDT) of 0.5 °C below a patient’s body temperature measured at the beginning of each haemodialysis session, compared to a standard dialysis temperature of 36.5 °C. To inform how to deploy the IDT across many haemodialysis centres, we assessed haemodialysis physicians’ and nurses’ perceived barriers and enablers to IDT use.

**Methods:**

We developed two topic guides using the Theoretical Domains Framework (TDF) to assess perceived barriers and enablers to IDT ordering and IDT setting (physician and nurse behaviours, respectively). We recruited a purposive sample of haemodialysis physicians and nurses from across Ontario and conducted in-person or telephone interviews. We used directed content analysis to double-code transcribed utterances into TDF domains, and inductive thematic analysis to develop themes.

**Results:**

We interviewed nine physicians and nine nurses from 11 Ontario haemodialysis centres. We identified seven themes of potential barriers and facilitators to implementing IDTs: (1) awareness of clinical guidelines and how IDT fits with local policies (knowledge; goals), (2) benefits and motivation to use IDT (beliefs about consequences; optimism; reinforcement; intention; goals), (3) alignment of IDTs with usual practice and roles (social/professional role and identity; nature of the behaviour; beliefs about capabilities), (4) thermometer availability/accuracy and dialysis machine characteristics (environmental context and resources), (5) impact on workload (beliefs about consequences; beliefs about capabilities), (6) patient comfort (behavioural regulation; beliefs about consequences; emotion), and (7) forgetting to prescribe or set IDT (memory, attention, decision making processes; emotion).

**Conclusions:**

There are anticipatable barriers to changing healthcare professionals’ behaviours to effectively deliver an intervention within a randomised clinical trial. A behaviour change framework can help to systematically identify such barriers to inform better delivery and evaluation of the treatment, therefore potentially increasing the fidelity of the intervention to increase the internal validity of the trial. These findings will be used to optimise the delivery of IDT in the MyTEMP trial and demonstrate how this approach can be used to plan intervention delivery in other clinical trials.

**Trial registration:**

ClinicalTrials.gov NCT02628366. Registered November 16 2015.

**Electronic supplementary material:**

The online version of this article (doi:10.1186/s13063-017-1965-9) contains supplementary material, which is available to authorized users.

## Background

Clinical trials often require healthcare providers to change their behaviours to deliver the experimental technology or therapy being evaluated. Failure to change provider behaviour could result in patients failing to receive the experimental treatment, undermining the internal validity of the trial [1, 2]. Prior to implementing some types of treatment, there is an opportunity for trialists to use insights from behavioural science to understand and address anticipatable barriers to changes in routine care [3–5]. Assessing potential barriers to the delivery of clinical interventions prior to their use in a trial is consistent with the feasibility/piloting stage in the Medical Research Council (MRC) framework for the development and evaluation of complex interventions [6]. This approach is also important in a learning health system framework, where the efficient integration of clinical trials into routine care is advocated to better align research with practice [7]. Here we report use of a generalizable behavioural approach to systematically understand the barriers and facilitators to using an intervention to be deployed in a large pragmatic multi-centre cluster-randomized controlled trial.

### The MyTEMP trial

Over 2 million people worldwide receive ongoing life-sustaining haemodialysis for end-stage kidney disease [8, 9]. Haemodialysis is an effective renal replacement therapy, yet its side effects can lead to short and long-term symptoms and health implications [10, 11]. Mortality rates in people on haemodialysis are up to 7.8 times higher than in the general population [12, 13] due to the illness and the impact of haemodialysis itself [3, 6]. Haemodialysis can lead to ischaemia to vital organs, which over time may cause significant damage [10, 11]. Intra-dialytic hypotension (≥20 mm Hg reduction in systolic blood pressure or a decrease in mean arterial pressure by 10 mmHg [14]) occurs in up to 30% of treatments and may contribute to the ischaemia of vital organs [10]. New treatment is needed to prevent ischaemia during dialysis. Using temperature-reduced haemodialysis is a promising approach yet to be trialed at scale for effectiveness [10, 15–18].

A systematic review of temperature-reduced haemodialysis identified 26 randomized controlled trials and showed that intra-dialytic hypotension occurred 70% less frequently in patients using temperature-reduced dialysis than in those on standard temperature dialysis [19]. However, the review highlighted a need for better evidence given that most trials were small and quality was low [19].

Implementing temperature-reduced dialysis has also been met with concerns about patient comfort [19–21]. A randomised crossover study of 10 patients compared the effect of dialysate temperatures of 37 °C and 35 °C on heart function, and assessed patient comfort. Three out of ten participants felt uncomfortable when the dialysate temperature was set at 35 °C, and two out of ten participants were able to detect the cooler dialysate temperature [20]. However, another study (10 patients) reported that after being dialysed at 35 °C for three sessions compared to 36.5 °C, most patients (*n* = 8) felt more energetic, reported better general health and wanted to continue receiving a cool dialysate temperature [21]. Out of 26 trials in the aforementioned review, 10 also reported on patient comfort, in which negative symptoms (feeling cold, shivering, or having cramps) were not statistically different between cooler and fixed dialysate temperatures [19]. A potential solution to address patient comfort involves individual tailoring of reduced dialysate temperatures to each patient’s core body temperature at the start of each dialysis session [15, 17].

Responding to calls for a larger-scale evaluation of individualized dialysate temperatures (IDTs) on major outcomes (i.e., heart attack, stroke, cardiovascular revascularization, and death), we are planning a large-scale pragmatic cluster trial (Major outcomes with personalized dialysate temperature (the MyTEMP trial); ClinicalTrials.gov: NCT02628366). In 2017, the MyTEMP trial will randomize 84 haemodialysis centres in Ontario, Canada to provide usual care (dialysate temperature in most Ontario centres is set at 36.5 °C for most patients) or IDTs (where the dialysis temperature will be set individually at 0.5 °C lower than the patients’ core body temperature measured prior to the start of each haemodialysis session). Therefore, in the trial treatment arm, haemodialysis physicians and nurses will need to change their current behaviours related to dialysate temperature prescribing and setting behaviours, respectively. Personalizing the dialysate temperature may seem like a relatively simple clinical action, but nevertheless represents a shift from routine practice across many centres. Identifying potential barriers to intervention delivery prior to testing the intervention in a trial may help to inform strategies to ensure uptake and maintain treatment delivery in participating trial centres [22–24].

### Behavioural diagnostics of barriers and facilitators to clinical behaviour change

Barriers to clinical behaviour change may be complex and involve multiple factors at multiple levels [25, 26]. Behavioural science has developed and evaluated a range of theories that may help explain behaviour and behaviour change in healthcare professionals. These theories can serve as a basis for investigating barriers and facilitators to implementing new or altering current behaviours. There have been calls for greater understanding about contextual factors in trials including personal, organizational, trial and the problem context [27]. When identifying factors that might impede clinical behaviour change, it can be useful to apply a comprehensive theoretical framework that summarizes constructs across a range of theories to ensure sufficient breadth of factors are explored [28, 29]. The Theoretical Domains Framework (TDF) synthesizes a wide range of psychological theories and constructs applicable to behaviour change [28, 30]. The TDF-1 encompasses 128 constructs and 33 psychological theories organized into 12 domains [28]. A validation study of TDF domains supported the original domain structure, and was refined into the TDF-2 by splitting three domains and removing one [30] resulting in 14 theoretical domains: knowledge; skills; social/ professional role and identity; beliefs about capabilities; beliefs about consequences; optimism; reinforcement; goals; intention; environmental context and resources; emotion; memory, attention and decision making; social influences; and behaviour regulation [28, 30]. The present study used domains in TDF-2, whilst including the nature of the behaviour domain from TDF-1.

The TDF has been applied to systematically and comprehensively assess the barriers and facilitators to a range of healthcare behaviours. It can be used to understand the factors that drive current practice, which may prevent the uptake of behaviours associated with evidence-based care [28, 29]. In its most common application, the TDF has been used to assess barriers and facilitators to increasing or decreasing a clinical behaviour for which a gap in care has been identified, to inform the development of an implementation intervention to be trialed [3, 5, 31]. For instance, Patey and colleagues [32] investigated anaesthesiologists’ and surgeons’ unnecessary ordering of pre-operative tests in low-risk patients and showed that they had conflicting views about test ordering responsibilities (social professional role and identity), anticipated and ordered tests based on other physicians’ preferences for test ordering (social influences) and did not cancel orders made by other physicians (beliefs about capabilities). Further, the SuDDICU study used the TDF to investigate barriers and enablers to the use of selective decontamination of the digestive tract [33]. Concerns about antibiotic resistance (beliefs about consequences) and low priority (motivation and goals) were the most extensively represented views across clinicians [34, 35]. A further sub-study with senior nurses highlighted lack of awareness of the procedure (knowledge), patient comfort (beliefs about consequences), costs to the organization (environmental context and resources) and competing priorities (motivational and goals) [36]. The TDF has also been used to investigate ways to improve an existing implementation strategy, e.g., optimising care for sepsis treatment [37]. In addition, the TDF has been used to understand why trials of interventions have not been effective. For instance a trial of a clinical decision tool for ordering computed tomography scans in cases of mild head injuries in the emergency department showed no effect [38]. Curran and colleagues [39] used the TDF to show that there remained a need for greater clarity on the use of the tool (beliefs about capabilities) and in the use of clinical judgment relative to the new tool (behaviour regulation) and remembering to use the tool (memory, attention, and decision-making). Such barriers may have been addressable as part of delivering the intervention in the trial, had they been identified prior to initiating the trial.

The use of qualitative approaches is increasingly common alongside trials, yet only in a minority have these been conducted prior to the trial [40]. To our knowledge, the TDF has not yet been used to investigate the barriers and facilitators to clinical behaviour change within the context of, and prior to, delivering an intervention in a multi-centre clinical trial primarily designed to evaluate a treatment’s effectiveness in improving patient outcomes. We investigated the barriers and enablers to physicians prescribing and nurses setting IDTs to inform a strategy to optimise the delivery of IDTs in multiple haemodialysis centres that will be randomly allocated to the intervention arm of the MyTEMP trial.

## Methods

### Participants

Haemodialysis temperature management typically involves two sequential clinical behaviours: a physician or nurse practitioner orders/prescribes the temperature then a registered nurse sets the temperature on the dialysis machine prior to each dialysis session. In Ontario, physicians are responsible for prescribing and nurses can have various roles that differ across centres. For example, nurse practitioners and primary care nurses in some centres have prescribing duties, and other nurses have administrative duties while overseeing frontline staff and have experience in providing the haemodialysis treatment. The inclusion criterion for the interviews was that participants must be haemodialysis physicians or nurses. Our planned sample size was informed by the 10 + 3 rule for demonstrating data saturation when using theory-based interviews [41].

### Recruitment and procedure

Participants were a purposive sample of physicians and nurses from haemodialysis centres in Ontario from prospective MyTEMP trial sites. Trial investigators identified potential participants from contact lists in each site and invited them by email to participate in an interview. Those interested contacted the study coordinator to arrange a time for the interview. We invited 18 physicians and 17 nurses across Ontario; 18 (from 11 different centres) agreed and were interviewed (9 physicians, 9 nurses), 2 declined, and the remainder did not respond. The majority of interviews were conducted by phone and two were conducted in person based on participant preference. BM, trained in psychology and implementation science-based approaches and not a member of the respondents’ professions (to promote open discussion), conducted all interviews. Interviews were audio recorded and transcribed.

### Interview topic guide development

Interview guides were designed to identify factors that may influence whether IDTs would be used for all patients in the trial at centres that were randomly allocated to an individualized temperature, consistent with the intervention arm of the MyTEMP trial. We first identified “*who* needs to do *what* differently?” [31]. Implementing IDTs in the treatment arm of the trial requires change in at least two sequential clinical actions: prescribing/ordering IDTs for all patients (a physician’s or nurse practitioner’s behaviour) and setting IDTs for all patients (a nurse’s behaviour). We described the behaviour in terms of the target, action, context, time, actor (TACT-A) principle [42]: target (who will be affected by the behaviour, i.e., all patients on dialysis); action (the observable behaviour itself; prescribing or setting); context (physical location in which the behaviour takes place; in the dialysis centre); time (when the behaviour occurs; for prescribing: prior to the patient arriving for each dialysis treatment; for setting: at the start of the treatment); and actor (the person who does the behaviour: physician or nurse practitioner, and nurse).

We developed similar but separate interview guides for prescribers (physicians and nurse practitioners) and those who set dialysate temperatures (nurses). Both guides were based on the TDF-2, with the domain “nature of the behavior” from TDF-1 added at the analysis phase [30]. Interviews were designed to elicit thoughts, beliefs and opinions on the barriers and facilitators to prescribing by a physician or setting IDTs by nursing staff for all patients. Most haemodialysis centres in Ontario use a standard dialysate temperature of 36.5 °C applied to all patients. Implementing the altered treatment in the MyTEMP intervention arm will involve an IDT being set for each patient in each treatment session. Accordingly, the interview guide specified a hypothetical behaviour not currently being performed. The behaviour was discussed in the guide within the context of implementing the MyTEMP trial.

Interview guides were drafted, refined, and piloted with members of the research team, then piloted with one nephrologist and one haemodialysis nurse to ensure length and applicability. The final topic guides are available as Additional files 1 and 2.

### Data analysis

Interviews were audio recorded, anonymised and transcribed verbatim. Analysis involved using NVivo 10 software (QSR International, 2012) and consisted of three steps: (1) coding utterances from the interviews into the TDF domains; (2) generating belief statements (representative descriptions of utterances across respondents) within domains; and (3) generating overall themes and sub-themes across all interviews and domains. Accordingly, the analysis involved a combination of directed content analysis [43] through coding utterances into TDF domains, and thematic analysis within and across domains [44].

### Codebook development and coding to TDF domains

We concurrently conducted interviews and coding. We iteratively developed a codebook to maintain coding rule transparency. Analysis was guided by TDF-2 [30]; however, when coding the first interview it became apparent that “nature of the behaviour” from TDF-1 [28] would help to capture relevant features of the behaviours of nephrologists and haemodialysis nurses. Clarifications and changes to the codebook were made during the course of the study based on consensus discussions between the coders as the analysis progressed (see Additional File 3 for the final code manual).

Six interviews were independently double coded (BM, JP) in blocks of three interviews. The first three were coded to update the codebook and the two coders compared coding. When discrepancies arose in coding text into a domain, the coders discussed until consensus was reached and the codebook was adapted to reflect changes. Coding single utterances to multiple TDF domains was permitted. Assessment of inter-rater agreement using Krippendorff’s alpha and kappa was conducted for the second block of coded interviews; a priori we planned that if agreement statistics did not reach formal thresholds (0.80), double coding would proceed in blocks of three until the threshold level of agreement was reached.

Belief statements were generated within each domain: utterances expressing similar content were grouped and assigned a representative summary statement [45]. Similar belief statements were then grouped together to form sub-themes. All belief statements and sub-themes were generated by one coder and verified by another. Belief statements and sub-themes were then used to develop representative overall themes, which were developed iteratively between the two coders and the rest of the research team.

Consistent with existing criteria [32], TDF domain relevance was assessed based on frequency with which content was coded at each domain, presence of conflicting belief statements, and particularly salient themes on the use of IDTs determined through discussion with the wider multidisciplinary research team, including clinical experts in nephrology, trials, health services research, implementation science, and health psychology.

## Results

### Participants

Interviews were conducted between December 2015 and April 2016 and lasted between 25 and 66 minutes (media*n* = 47 minutes). Nine physicians (two women) and nine nurses (nine women) participated in interviews, from 11 dialysis centres across Ontario (7 Eastern Ontario; 1 Northern Ontario; 10 Southern Ontario) in academic [10] and non-academic settings [8]. The median number of years in the job reported at interview was 15 years (range from 6 months to 40 years).

### Inter-rater reliability

Data from physicians and nurses were analysed together and consisted of 163 belief statements across 15 domains. Krippendorff and kappa inter-rater agreement scores for three interviews were both 0.82 (second block of transcripts), indicating agreement and that interviews could be reliably coded into respective domains [46].

### Overall themes and TDF domains for setting and prescribing IDTs

TDF interviews with haemodialysis physicians and nurses led to the identification of seven themes. Tables 1 and 2 summarize barriers and facilitators (respectively) in terms of the themes, sub-themes, underlying beliefs and associated TDF domains, and the number of interviewees describing each. The following identifies representative quotes for each theme.

**Table 1:** Potential barriers to prescribing and setting individualized dialysate temperatures (IDTs) (*n* = 18)

Themes	Sub-theme	Belief statement	Frequency (out of 18)	Theoretical Domains Framework, domain(s)
Theme 1: awareness of clinical guidelines and how IDT fits with local policies	Awareness of guidelines	- We don't use guidelines for individualized cooler dialysate temperatures	14	Knowledge
		- There are no guidelines for dialysate temperature	5	
	Potential for conflict of IDT with local policies	- Individualized cooler dialysate temperatures will/may conflict with local policies	3	Knowledge/Goals
Theme 2: benefits and motivation to use IDT	Not a priority	- It’s a little priority at this point	10	Goals
	No rewards in place	- I can’t think of any rewards	8	Reinforcement
	Motivation limited to subset of patients	- I am more motivated to set or prescribe cooler dialyste temperatures when my patients have hypotension on dialysis	6	Intention
		- I am not inclined to use individualized cooler dialysate temperatures for patients doing well on current dialysate temperatures	5	
		- You have to weigh the benefits of preventing hypotension with patient complaints of feeling cold	3	
Theme 3: IDT alignment with usual prescribing and setting practices and roles	Currently not individualizing dialysate temperatures at each treatment	- We don't individualize dialysate temperatures	10	Nature of the Behaviour
		- When setting or prescribing cooler dialysate temperatures it is usually 0.5 degrees below standard	8	
	Sometimes individualize the dialysate temperature	- I occasionally or rarely prescribe or set cooler dialysate temperatures	11	Social Professional Role and Identity/Nature of the Behaviour/ Beliefs about Capabilities
	Nurses require physicians' order for permanent change in dialysate temperature	- We need a global order/ policy change/ medical directive so nurses can set individualized cooler dialysate temperatures	7	Social Professional Role and Identity/Social Influences
		- We would need a doctor's order to set individualized cooler dialysate temperatures	5	
		- I need an order from the doctor for a permanent change in dialysate temperature beyond one treatment session	3	
Theme 4: thermometer availability/ accuracy and dialysis machine characteristics	Outdoor temperature and drinks can influence temperature reading	- Climate in winter or summer can impact accuracy of core body temperature readings	3	Environmental Context and Resources
		- Consumption of warm beverages or ice can impact accuracy of core body temperature readings	3	
	Thermometer availability	- Potential limited thermometer availability	2	Environmental Context and Resources
	Dialysis machine can be adjusted in 0.5 or 0.1 increments up to 35 degrees Celsius	- Can adjust dialysate temperatures by 0.5 increments	2	Environmental Context and Resources
Theme 5: impact on workload	Negative impact on workload	- Physicians say nurses’ workload will increase	6	Beliefs about Capabilities/ Beliefs about Consequences
		- My workload will increase	4	
Theme 6: patient comfort	Negative clinical management consequences	- Patients may feel too cold on cooler dialysate temperatures	11	Beliefs about Consequences
		- It is common for patients to feel cold on dialysis	7	
	Coping plans that lead to increased dialysate temperature	- If patients are really complaining of being cold we may increase dialysate temperature by 0.5	9	Behavioural Regulation
		- I may increase the dialysate temperature for someone with hypertension to see if that decreases their blood pressure	2	
		- If patients are feeling cold and have no issues with blood pressure or fever and request an increase in dialysate temperature I would not have evidence to deny their request	2	
	Emotions related to patient comfort	- I may feel worried or concerned if patients are feeling cold	6	Emotion
Theme 7: forgetting to prescribe or set IDT	Potential to forget	- I may forget to prescribe or set an IDT if I am busy	9	Memory, Attention and Decision Making /Emotion
		- We would need reminders for IDTs	6	
		- It may be easy to forget in emotional or tense situations	2

**Table 2:** Potential facilitators to prescribing and setting individualized dialysate temperatures (IDTs) (*n* = 18)

Theme	Sub-theme	Belief statement	Frequency (out of 18)	Theoretical Domains Framework, domain(s)
Theme 1: awareness of clinical guidelines and how IDT fits with local policies	Awareness of need for more evidence	- It needs to be studied	18	Knowledge
	Awareness of evidence	- An intervention that’s been studied for which there’s reasonable evidence of benefit	10	
	Awareness of guidelines	- There are guidelines for dialysis treatment	7	
	Link with existing policies	- Individualized cooler dialysate temperatures will not conflict with local policies	12	Goals
	Centres have existing temperature standards	- Centre standard is 36.5 or higher	10	Knowledge/Goals
		- Centre standard is less than 36.5	6	
Theme 2: benefits and motivation to use IDT	Positive clinical management consequences	- Cooler dialysate temperatures can help manage or prevent hypotension during dialysis	17	Beliefs about Consequences
		- Cooler dialysate temperatures can help with fluid removal during dialysis	7	
	Positive potential long-term consequences	- Individualized cooler dialysate temperature may lead to better cardiovascular outcomes	8	Beliefs about Consequences
		- Individualized cooler dialysate temperatures may lead to a reduction in morbidity and mortality or increase longevity	3	
		- Individualized cooler dialysate temperatures may preserve cognitive function	2	
	Optimistic	- Based on what I'm hearing, I'm quite optimistic	16	Optimism
	Patient benefit is inherently reinforcing	- If you can prevent symptomatic hypotension for your patients, that’s rewarding	11	Reinforcement
	Priority	Setting/prescribing IDTs is a priority because we need to know the answer	7	Goals
Theme 3: IDT alignment with usual prescribing and setting practices and roles	Procedures and roles specific to physicians	- The physician would order or prescribe individualized cooler dialysate temperatures	14	Social Professional Role and Identity/Nature of the Behaviour/Beliefs about Capabilities
		- Physicians are responsible for prescribing dialysate temperatures	11	
		- Prescriptions are applicable over all treatments until changed again	8	
		- I would have to be able to prescribe IDTs in a way that I wouldn't have to review every treatment because that would not work	5	
	Procedures and roles specific to nurses	- We usually measure core body temperature before and after treatment	8	Social Professional Role and Identity/Nature of the Behaviour/Beliefs about Capabilities
		- Nurses can modify dialysate temperature during treatment	5	
		- Dialysate temperature is set automatically or is a default	5	
		- We usually accept treatment parameters	3	
	Influences among health care professionals	- Nurses follow the doctor's orders or prescription	12	Social Professional Role and Identity/Nature of the Behaviour/Beliefs about Capabilities
		- Nurses influence physicians when prescribing dialysate temperature	10	
	It will be easy to prescribe or set IDTs	- I am confident that I will be able to prescribe IDTs for all my patients	10	Beliefs about Capabilities
		- It will be easy to set individualized cooler dialysate temperatures	8	
		- It will be easy to prescribed IDTs	8	
Theme 4: thermometer availability/accuracy and dialysis machine characteristics	Dialysis machine can be adjusted in 0.5 or 0.1 increments up to 35 Celsius	- Can adjust dialysate temperature by 0.1 increments	3	Environmental Context and Resources
Theme 5: Impact on workload	Impact on workload	- My workload will increase minimally	10	Beliefs about Capabilities/Beliefs about Consequences
		- Reducing episodes of hypotension during dialysis can decrease workload	7	
		- My workload will not increase	6	
Theme 6: patient comfort	Tolerability	- Patients are not likely to notice the cooler temperature/not likely to be side effects/generally well-tolerated	8	Beliefs about Consequences
	Coping plans for patients who say they are cold	- We give blankets to patients who feel cold on dialysis	12	Behavioural Regulation
		- For patients who feel cold on dialysis, we suggest that they wear warm clothing and bring blankets	5	
	No emotion related to IDTs	- I don’t or I won’t have any emotions related to dialysate temperature	6	Emotion
Theme 7: forgetting to prescribe or set IDT	Unlikely to forget	- I won’t forget	7	Memory, attention and decision making

Theme 1: Awareness of clinical guidelines and how IDT fits with local policies

*Centres have existing temperature standards*: nearly all participants described having a centre standard dialysate temperature, where the majority of patients had a static dialysate temperature set at every treatment session, unless another dialysate temperature had been prescribed for clinical reasons. Standard dialysate temperatures differed between centres with the majority of participants (10 out of 18) reporting a standard protocol of 36.5 °C.

*Awareness of guidelines*: five physicians stated that they were unaware of specific guidelines or centre policies for dialysate temperature and some noted that the dialysate temperatures prescribed could differ between dialysis centres.

To my knowledge, there are no guidelines that recommend a given temperature. There are certainly practices that apparently vary from place to place. (Physician #9)

Similarly, the majority of participants (8 physicians, 6 nurses) reported that there were no guidelines for individualized dialysate temperatures.

Do you use any guideline recommendations for prescribing individualized cooler dialysate temperatures? (Interviewer)

I mean this initiative is so new. I’m trying to think if there’s a hard [guideline] at the moment. A lot of the work that are nephrology research is all done - is all published in scientific journals. I know there’s discussion about the temperature. I don’t think there’s a guideline per se about it. (Physician #18)

*Potential conflict of IDT with local policy*: the majority of participants did not foresee using IDTs as conflicting with local policies; however, three nurses reported that their local policy would have to be changed to implement the setting and prescribing of IDTs.

Well, currently we have a standard temperature so it is in conflict with that so that would have to be altered. (Nurse #9)

*Awareness of need for better evidence:* all participants agreed that having better evidence and more study of the effectiveness of IDT is needed.

Theme 2: benefits and motivation to use IDT

*Positive clinical management consequences*: most (9 physicians, 8 nurses) believed that cooler dialysate temperatures would benefit specific patients, mainly those at high risk for intra-dialytic hypotension. Two nephrologists and five nurses reported that a lower haemodialysis temperature would assist with fluid removal during dialysis treatment.

If the patient is becoming more hypotensive or you see a decline in systolic pressures, we will decrease the temp within reason in the hopes of, obviously, vasoconstriction to help maintain the blood pressure as we pull fluid from the vascular space. (Nurse #11)

*Positive potential long term consequences*: four physicians and four nurses reported potential long-term benefits for all patients as a result of using IDTs, including increasing longevity, better cardiovascular outcomes, and the fact that cooler dialysate temperatures may help to preserve cognitive function.

*Motivation limited to subset of patients*: three physicians and two nurses said they were less inclined to set or prescribe an IDT if patients are doing well on the current temperature.

You might go “Wait a minute. Why does everybody have to be cold if only 30% of people are going to drop their blood pressure?” (Physician #6)

Well, if the patient is okay with the set temperature, I wouldn’t touch anything. (Nurse #1)

Theme 3: IDT alignment with usual prescribing and setting practices and roles

**Fig. 1:**
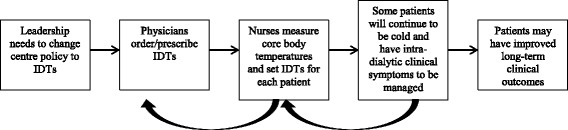
Process of who needs to do what differently, inter-relationships and outcomes. In the MyTEMP trial, the leadership at each dialysis centre should change local policy to ensure alignment with individualized dialysis temperatures (IDTs). Physicians should order IDTs for current patients at one time, and as new patients receive prescriptions for dialysate temperature. Nurses are likely to follow physician orders to set IDTs. Nurses will be aware of patient feedback and other clinical symptoms related to IDTs. If changes need to be made to dialysate temperature prescriptions, nurses will likely inform the physician. Finally, patients may experience improved clinical outcomes as a result of IDTs

It was clear that physicians prescribe dialysate temperatures (eight physicians), and nurses set dialysate temperatures (nine nurses). However, there may be complex role and social influences on the process of using IDTs for *all* patients, particularly with respect to the interactions between physicians, nurses, and patients, which may need to be addressed (see Fig. 1).

*Procedures and roles specific to physicians:* physicians will need to change their prescribing behaviour to conform to the trial’s definition of IDTs (0.5 °C below each patient’s measured core body temperature) rather than their existing behaviour of prescribing a static temperature (e.g., 36.5 °C). Physicians reported that they usually prescribe a dialysate temperature once when a patient first starts receiving dialysis treatment and that the dialysate temperature prescription is applicable to all treatments going forward until changed again.

These dialysis orders are recurrent, meaning that they are valid until there’s another change. If there’s a change to another parameter then all of the other parameters stay the same. I could change one day the temperature. In three days if I change the potassium or the temperature, it’s going to remain whatever it was set at. (Physician #1)

Four physicians and one nurse practitioner noted that if the MyTEMP trial required them to prescribe an IDT for every patient in every treatment session, this would pose a significant barrier to trial implementation.

I’m not going to have nurses call me with the temperature and write the temperatures of all the patients. That would never fly. Okay. You will get too many pages. […] So, it will have to be standard. It will have to be core temperature is this, subtract that much and that’s the dialysis temperature with some parameters. (Physician #7).

If I have to write an order on every patient every time, it would influence my workload tremendously. I would not have the time and I think it would falter and not order it. (Nurse with prescribing role #9)

As a result, four physicians and one nurse practitioner reported that they would prescribe IDTs to all patients at one time. This is consistent with standards already in place for individualizing the amount of fluid removed.

You just do it once and say that the dialysate temperature is supposed to be 0.5 less than their core temperature and that would stand forever until I discontinued it. (Physician #6)

*Procedures and roles specific to nurses:* rather than setting a prescribed static dialysate temperature for each patient, nurses will have to set the IDTs for each patient by subtracting 0.5 °C from the measured core body temperature, every treatment session. Nurses described many existing procedures for setting dialysate temperatures: manually entering prescribed temperatures for all patients every treatment session; using pre-programmed cards; and software that automatically sets the dialysate temperature upon verification by the nurse. Whilst five nurses described having some autonomy to modify dialysate temperatures for the current dialysis treatment session if indicated due to patient symptoms, others noted that they do not modify dialysate temperatures without a physician’s prescription. Additionally, permanent changes to dialysate temperatures require an order from the doctor (reported by three nurses).

They would because they (nurses) can change it manually for that treatment but if they decided this patient today, I’m going to drop them to 35.5, they can do it for that treatment but unless they go in and change that patient’s order in [Dialysis charting software], the next treatment, they would come back in at 36.5. (Nurse #5)

*Dialysate temperatures are not currently individualized each and every treatment:* no participant-individualized dialysate temperature based on core body temperature each treatment:

The individualized in every treatment is very different from what I’ve ever done. (Nurse #5)

Once they have their first treatment, we have memory cards that memorize like codes data for the treatment. So we set it initially and then we wouldn’t change it unless we had an order to change the temperature. So once you put the card in and if you accept all the treatment parameters, usually we’re not changing it. (Nurse #10)

Some reported using cooler dialysate temperatures based on preference and patient clinical factors indicating that a cooler temperature would be beneficial for a particular patient. When cooler temperatures are prescribed, consistent with current recommended protocol for adjusting dialysate temperatures, they tend to be 0.5 °C cooler than the standard or the previously prescribed temperature, rather than based on core body temperature (eight participants).

There’s not a lot of people who actually look at the core patient’s temperature to decide what they’re going to set the dialysis temperature. It’s more, let’s say patients having recurrent low blood pressure. You look at their prescription. They’re at 36.5. You’ll say let’s drop it to 36. We’re not going to look at what their core temperature is. We’re just going to say, “Let’s drop them by a .5 or a one degree towards the cooler side.” (Physician #1)

*Influences among healthcare professionals:* participants described how nurses spend more time with patients and have an important role of informing physicians of symptoms that may influence their prescribing decisions (six physicians, four nurses).

A nurse, usually because our nurses will interact a lot more with our patients on a day-to-day basis than we do so a nurse may suggest, oh, Mr. So and so is feeling cold. Do you mind raising the temperature or vice versa? Do you mind decreasing the temperature? We might say, “Yes, sure. That’s a good idea. Let’s do it.” (Physician #1)

I think you’re always in conjunction with your doctor, right? If you see this as something that chronically needs to be ordered and brought forward, definitely a team, and of course you do sometimes seek others opinions or give reports saying “This is what I’ve done,” but primarily it’s the nurse and in conjunction with the doctor, if you see that is being a permanent change. (Nurse #11)

*Nurses require physicians’ order for permanent change in dialysate temperature:* five physicians and two nurses perceived that a medical directive to standardize IDTs in the centre or a “blanket order” applicable to all patients would be required to implement the MyTEMP trial. This would allow nurses to modify dialysate temperatures for each patient every treatment session as would be required for the MyTEMP trial.

Yes. So it would just automatically be done because they have protocols in place for so many other things like what their potassium number is, often there's a protocol in place where the nurses will change the dialysate potassium without notifying the physician. So it's something like that where the physician could be bypassed. (Physician #13)

Rather than setting a prescribed static dialysate temperature for each patient, nurses will have to set the IDTs for each patient by subtracting 0.5 °C from the measured core body temperature, every treatment session. Whilst five nurses described having some autonomy to modify dialysate temperatures for the current dialysis treatment session if indicated due to patient symptoms, others noted that they do not modify dialysate temperatures without a physician’s prescription.

No, we’re not playing around with the temperature. (Nurse #10).

Additionally, permanent changes to dialysate temperatures require an order from the doctor (reported by three nurses).

They would because they (nurses) can change it manually for that treatment but if they decided this patient today, I’m going to drop them to 35.5, they can do it for that treatment but unless they go in and change that patient’s order in [Dialysis charting software], the next treatment, they would come back in at 36.5. (Nurse #5)

Further, six physicians and nine nurses stated that nurses require and follow physician orders or prescriptions and five nurses said they would need a physician’s order or prescription to set IDTs for their patients if they were required to do so in the MyTEMP trial.

I don’t think I would do it on my own. I would still need the doctor’s permission or his or their knowledge that I’m doing it. I am not authorized to change the temperature ad lib, so to speak. (Nurse #6)

Theme 4: thermometer availability/accuracy and dialysis machine characteristics

Typically, body temperature is measured but does not impact inputting of dialysate temperature. However, the centres that will be randomized to the intervention arm of the MyTEMP trial *must* measure core temperature *before* the patient starts treatment so that they can set the IDT. Two issues were noted concerning using core body temperature to determine a unique dialysate temperature for each patient every treatment session.

*Thermometer availability*: in usual practice, nurses set dialysate temperatures prior to the start of dialysis treatment, either by verifying the automatic setting of dialysate temperatures or setting the dialysate temperatures manually. Eight nurses reported that they measure patients’ core body temperatures pre-dialysis and post-dialysis, and two nurses expressed concern about the possibility that the lack of thermometers at the centre may interfere with the timing of setting dialysate temperature at the beginning of treatment. A limited number of thermometers may be a barrier if nurses must wait for a thermometer to become available.

It is when we have an adequate amount of thermometers around. Right now we do but every once in a while one may go down and sometimes two. In which case, staff is doing more running around the unit looking for available thermometers. I think the availability and the number of thermometers that are available close to where the patients are may impact as well people taking temperatures. (Nurse #8)

*Outdoor temperature and drinks can influence temperature reading*: one physician and two nurses expressed concern about core temperature reading accuracy based on season and outdoor temperature (i.e., cold Canadian winter or hot summer) and how this may impact the accuracy of core temperature readings when patients first come to the dialysis centre from the outdoors.

Yes because they come in from the cold and you might not even want to set it because it might be quite cold, but an hour later. They’ve got blankets on and they’re warming up a little bit. Even a dialysate temp at 36 might be warming them up compared to what they are when they came in. Do we set them? Keep them that cold for the whole run or do we do temperatures hourly or an hour later and then set it? (Nurse #8).

Three nurses also flagged that when patients chew ice or drink warm beverages, the accuracy of core temperature readings may be compromised when oral thermometers are used.

What I see as a potential difficulty of the study is a number of our patients miss out in their pre-dialysis temperature because they’re already chewing on ice as they come in the door. So we’re going to have to try and figure out how we can get around that. So we do get a pre-dialysis temperature on everybody. (Nurse #7)

In addition, some dialysis machines had the capability to reduce the temperature in increments of 0.1 degree, while machines in other centres operated in increments of 0.5 degrees.

I can put whatever. It doesn’t have to be 0.5 degrees; it could be 0.7 degrees, 0.8 degrees. (Nurse #6)

With these machines, the temperature you can only change it by 0.5. So if you had to do it like anything other - if you had to deal something like 36.8 and then make it 0.5 less, these machines won’t allow you to do that. (Nurse #10)

Theme 5: impact on workload

*Workload as an enabler*: the majority of participants viewed prescribing and setting IDTs as an easy behavior (eight physicians, eight nurses). All physicians and one nurse practitioner described being confident that they would be able to prescribe IDTs to all patients under their care. Physicians described minimal to no increase in their workload if IDTs and the MyTEMP trial was implemented. Four nurses described that setting IDTs would increase their workload, particularly at the beginning of the MyTEMP trial. However, five nurses anticipated that IDTs would have minimal to no impact on their workload overall (including two nurses who anticipate an increase in workload at the beginning of implementation).

It would be a change of practice. It would be like in any other change in practice - you know first of all, making sure your temperature is accurate, the first one actually going through the steps in it. […] It would probably put another minute of work in putting somebody on. (Nurse #10)

Four physicians and three nurses described how IDTs may decrease their workload, if the cooler dialysate temperatures lead to a reduction in the incidence of intra-dialytic hypotension.

If patients actually have fewer hypotensive episodes and really those are ones that were - if you’re talking from a unit perspective, those would be symptomatic hypotensive episodes that require intervention. From a unit perspective, if you have less symptomatic hypotension, then you have [to] intervene less, that’s less work. (Physician #6)

Theme 6: patient comfort

*Negative clinical management consequences*: many participants were concerned about patient comfort, anticipating that patients will feel too cold with an IDT (three physicians, eight nurses).

It would be like being in an air conditioned room with not a lot of clothes on […] and sitting there for four hours. It’s not like it’s just a short period of time, it’s a long period of time. (Nurse #10)

One physician and six nurses noted that patients tend to feel cold on dialysis even if they are not receiving a “cool” dialysate temperature.

Most patients feel cold on dialysis irrespective of what temperature you’re giving them. (Physician #1)

Some of them - the patients are always cold. It’s an ongoing problem. Even when their temperatures are normal they still feel cold so you definitely need to have some patient buy-in if you’re deliberately freezing them. (Nurse #12)

*Emotions related to patient comfort*: eight nurses described potential negative emotions they may have if they were to prescribe or set an IDT. Most commonly, concern about patient comfort was identified as a potential influence on the decision to prescribe or set an IDT.

I worry about the people. I think that cute, little old lady’s already cold. I think I would feel a bit conflicted in doing that to her. (Nurse #12)

*Tolerability*: four physicians and four nurses thought that patients will be unlikely to notice the “cool” dialysate temperature as it will only be 0.5 °C below their core body temperature.

Some patients might find it cold but with the 0.5 degrees that we’re talking about for this study, that’s less likely to occur. Generally well-tolerated so I’m not very concerned. (Physician #10)

*Coping plans for people who say they are cold*: as participants described that patients tend to feel cold during treatment, 17 described strategies currently used to help alleviate these symptoms of feeling cold (i.e., blankets, extra clothing, etc.).

That would be the kind of advice that we would give is to maybe wear warm socks and warm clothing. The rest we would supply with blankets. (Nurse #7)

Participants also described instances when they would increase dialysate temperatures, namely: to help manage clinical symptoms such as high blood pressure during haemodialysis treatment (two physicians) and when patients reported feeling uncomfortably cold (six physicians, five nurses)

But there are some people who will complain of feeling cold and occasionally request warmer temperature. If they did not have problems with low blood pressure, I would not have enough evidence to deny their request. (Physician #9)

Theme 7: forgetting to prescribe or set IDTs

While two physicians stated they would not forget to prescribe an IDT, and three physicians thought forgetting would not be an issue as they would only have to prescribe IDTs at one time, six physicians and eight nurses identified potential reasons they might forget to prescribe or set an IDT (e.g., if the centre was busy, or if they became flustered in a particular situation).

I suppose the scenario where you just happen to hit a week where you’re particularly busy with patient load that you get distracted from this prescription concept. (Physician #12)

Then you forget to do things because you’ve got a patient saying, “I’m late being connected and my ride is at such and such a time” a lot of anxieties that the patient can put on the staff at times. So I think that could be a factor in it being forgotten. (Nurse #7)

Well number one, people may actually forget to make the change, I guess. I mean intense situations; emotional situations could be for other reasons, right? So, people could forget I suppose. (Physician #18)

## Discussion

We used the TDF to identify potential barriers and enablers to using IDTs with patients within haemodialysis centres, to design a strategy to deploy an intervention in an upcoming large-scale multi-centre clinical trial. The study identified seven themes of barriers and enablers that can directly inform use of IDTs in the MyTEMP clinical trial and promote greater fidelity of the delivered treatment in the intervention arm. To our knowledge this is among the first applications of the TDF to inform an understanding of barriers and enablers to clinical behaviour change involved in delivering the treatment in a planned clinical trial. While the “feasibility/piloting” phase of the UK MRC framework for developing and evaluating complex interventions often focuses on recipients of the intervention, the present study highlights the utility of understanding the perspective of those delivering the intervention within a clinical trial as well. The present study demonstrates the potential utility of conducting pre-trial behavioural diagnostics when intervention delivery depends on existing clinical staff (rather than a research team) altering their existing behaviours (as opposed to introducing a new treatment). This study may serve as a useful exemplar of how such an approach could inform other future clinical trial development.

While at face value, prescribing and setting IDTs are seemingly simple clinical actions we nevertheless identified a range of potential barriers and enablers that can directly inform the trial team to optimise use of IDT in the MyTEMP trial, including: the need for alignment (and staff awareness of such alignment) between local policies and the IDT protocol; awareness of resulting changes required to roles and usual practices; the potential that the trial could generate the evidence sought by healthcare professionals to justify its use in routine care; clarity that the potential benefits of IDT extend to all patients; the need for thermometer availability at the start of each patient’s dialysis session; the need to emphasise the potential positive impact on (reduced) workload; clarity on how to manage patient comfort in ways that respect patients while not undermining the intervention’s mechanism of effect; and the need to ensure that this change in routine practice remains salient to avoid forgetting.

### Implications for optimizing the implementation of IDTs in the intervention arm

#### A need to consider variations in usual procedures across centres

Across the centres there appeared to be differences in procedures for setting and prescribing dialysate temperatures, differing levels of autonomy amongst nurses for making adjustments to dialysate temperatures, and differences in the dialysate temperatures routinely used. These findings are consistent with a brief review discussing pragmatic clinical trials for chronic kidney disease, where deBoer and colleagues [47] highlighted potential sources of variation across centres in terms of procedures, differences in staff at the centres, and type of electronic health records. The findings are also consistent with suggestions for identifying barriers and developing tailored approaches to implementation of the IDT at each centre [4].

#### Need to address potential lack of motivation to use IDTs with all patients

The lack of awareness about the potential benefits of IDTs to all patients (and not just those with intra-dialytic hypotension, who may only be a minority of patients in some settings), and the belief that patients will feel too cold with IDTs, are important barriers that emerged from the analysis. The MyTEMP trial will itself address the lack of awareness about the potential benefits of IDTs to *all* patients. Most clinicians were aware that cooler dialysate temperatures can help to manage intra-dialytic hypotension, and some had used dialysate temperatures cooler than the centre standard to manage hypotension. However, while some were motivated to use IDTs for patients with hypotension, others were less inclined to use IDTs for patients “doing well” on the current dialysate temperature. This is consistent with trial literature on equipoise regarding physicians’ views and preferences about the superiority of one clinical approach versus another [48]. The trial implementation strategy should strengthen beliefs about consequences and motivation to use IDTs for *all* patients, including those doing well on a particular dialysate temperature.

Some participants thought that patients may feel too cold on IDTs, and this concern has been evaluated in previous studies investigating temperature-reduced haemodialysis [19, 20]. Most patients who have been on temperature-reduced haemodialysis report positive views of the experience and report wanting to continue to use the temperature [21]. While participants’ concerns about the IDTs are equivocal in the literature, the MyTEMP trial will use individualized cooler dialysate temperatures for each patient, rather than one cool temperature for all patients, in order to attempt to enhance tolerability for more patients [15]. Body temperature does not fluctuate greatly, and even small changes in body temperature can lead to shivering (i.e., 0.3 °C and 0.8 °C separate the shivering and vasodilation thresholds) [49]. The IDT will be 0.5 °C below core body temperature, so the actual IDT may not reach the threshold for shivering, therefore some patients will not notice the change in dialysate temperature. However, patient tolerability to IDTs and consideration of appropriate ways to alleviate symptoms of feeling cold (e.g., using blankets, clothing) without interfering with the mechanism of the intervention will be important to consider for the trial.

#### Need to address concerns about thermometer accuracy

Concerns about the realities of the Canadian climate and consumption of cold or warm beverages on core temperature readings taken via tympanic or oral thermometers need to be addressed. If it is −30 °C in the winter and a patient has a tympanic temperature taken, the measured temperature may be low, and subtracting 0.5 °C from that low temperature to set the IDT may be problematic. Similarly, taking the core body temperature orally too soon after a hot coffee or ice water may also lead to a high or low thermometer reading, leading to an IDT that inaccurately reflects core body temperature. Oral and tympanic measures of body temperature can significantly change from baseline measures of core body temperature after exposure to hot (43.5 °C) and cold (−5 °C) environments for up to 20 minutes after being exposed to such temperatures [50]. Consumption of hot water and ice water were found to impact oral thermometer readings for up to 9 minutes, but had no significant impact on the tympanic thermometer readings [51]. These potential influences on the measurement of core body temperature readings, and importantly, how to manage these issues, should be made clear to clinical staff in the intervention arm.

#### Sequences of clinical behaviours

Physicians’ actions have a clear impact on the behaviour of nurses, and nurses are influenced by patient comfort and are often the first to know of complications on dialysis, such as intra-dialytic hypotension. The nurse in turn has a clear influence on physicians’ actions by informing them of the patient’s clinical issues, which then influences physician’s prescribing behaviour (see Fig. 1). Accordingly, physicians and nurses frequently discussed each other’s roles during the interviews. Investigating multiple behaviours from multiple health professionals (Fig. 1) provided particular insight into barriers related to the domains of Social/professional role and identity and Social influences. In particular the behaviour of one profession, defined by their professional role, serves as a social influence to the other profession. Strategies for implementing the treatment should account for these roles and influences by clarifying the inter-personal processes within the protocol itself to better ensure that the required sequences are performed.

The implications for optimising the trial implementation were considered and strategies for addressing identified barriers and facilitators were proposed and discussed amongst the research team. A final set of 10 recommended strategies is presented in Additional file 4.

### Strengths and limitations

A strength of the study was that both physicians and nurses were interviewed. Possible limitations include that those who consented to participate may have been more in favour of using IDTs, though all dialysis centres in Ontario have agreed to participate in the MyTEMP trial, and believe IDT is an intervention worth testing. Nevertheless, barriers were identified and may inform the wider trial implementation strategy. By the nature of the investigation, the target behaviours were hypothetical; therefore unforeseen barriers may arise once the trial is in place despite an attempt to pre-emptively address important barriers. However, the behaviour is not completely new but rather a shift in a practice that shares many commonalities with usual care.

## Conclusion

A growing body of evidence supports the utility of using the TDF as a basis for identification of potential barriers and enablers to changing healthcare professionals’ behaviours [4, 31]. This literature has predominantly been developed within the field of implementation science, focusing on supporting healthcare professionals to implement evidence and clinical guidelines in routine care [3, 5, 37]. In such cases, the outcomes of interest tend to be patient care rather than patient clinical outcomes. The approach used in the present study is a novel and potentially a helpful methodological advance in the design and delivery of multi-centre clinical trials. When a clinical trial is not delivered by research staff, and instead involves a change in routine healthcare practices in the intervention arm, healthcare professionals must change their clinical behaviours for the duration of the trial. The fidelity of delivery of the clinical intervention and the findings of the trial depend upon their consistent altered practice and adoption of the new behaviour [1], which may be impacted by barriers and enablers to providing the trialed treatment [22, 23].

Even seemingly simple clinical behaviours may have unforeseen barriers that could be systematically assessed and addressed prior to the roll out of the trial [40, 52]. The TDF provides a theoretical framework rooted in behavioural science, representing a comprehensive set of factors related to behaviour change that can be interrogated to identify potential barriers and enablers. The methodological approach can be adapted to test other interventions beyond IDT. The result can help to identify and support or refute barriers and enablers that the trial team already suspects may influence the delivery of the intervention, and importantly, may identify factors previously not anticipated, as was the MyTEMP trial experience. Clinical trial development teams should consider adopting these approaches.

## Additional files

Additional file 1: Interview guide nurses. (PDF 109 kb)

Additional file 2: Interview guide physicians. (PDF 98 kb)

Additional file 3: Final coding manual. (PDF 244 kb)

Additional file 4: Proposed strategies. (PDF 114 kb)

## Abbreviations

IDT: Individualized dialysis temperature
MRC: Medical Research Council
MyTEMP: Major outcomes with personalized dialysate temperature
TACT-A: Target, action, context, time - actor
TDF: Theoretical Domains Framework
