# Supplementary material for: Barriers and facilitators to healthcare professional behaviour change in clinical trials using the Theoretical Domains Framework: a case study of a trial of individualized temperature-reduced haemodialysis
Source: Trials. 2017 May 22;18:227. doi: 10.1186/s13063-017-1965-9 (PMC5440991; doi:10.1186/s13063-017-1965-9)
Supplement: Supplementary file 1 — Interview guide nurses. (PDF 109 kb) [file 13063_2017_1965_MOESM1_ESM.pdf]

Interview Guide - Nurses

Explanation:

- Thank you for agreeing to speak with me about using cooler dialysate temperatures
- Approximately 30-45 minutes – audio-recorded
- Free to not answer, withdraw at any time
- Anonymised transcripts
- May be overlap, some answers repeated ok
- Your input as a nurse on the dialysis unit is very useful as we're trying to determine the best way to do a study on the effects of setting cooler dialysate temperatures on patient outcomes. So you know, my background is in psychology and I'm not a clinician. I'm interested in your views, experience and insight into the use of cooler dialysis temperatures. There are no right or wrong answers, and I'll probably ask you for clarification throughout our discussion.
- Any questions before we start?

Background of Informant

- Male or female (to keep track of, won't be asked)
- Confirm if right campus documented

1. What is your job title?
2. How long have you been a (job title)?

All of the following questions will concern the use of cooler dialysate temperatures. In relation to that, I would like you to think of the following specific activity:

Setting the dialysate temperature to 0.5^{o}C cooler than the patient's core temperature. We will refer to this as individualized cooler dialysate temperature.

Note: This individualized cooler dialysate temperature will be unique to each patient's treatment session. The individualized cooler dialysate temperature will be based on each patient's core body temperature at each dialysis treatment session.

If at any point you would like me to repeat this activity, please let me know.

To start, I'd like to get an idea of your usual procedure for giving dialysis treatment, and whether setting individualized cooler dialysate temperatures for the patients under your care is something that you ever do?

3. Do you normally check the core temperature of a patient?
    - Prompt: When?
4. Do you set the temperature of the dialysis machine?
    - If yes, Prompt: When
    - If yes, Prompt: what temperature do you usually set it at? (e.g. static or variable based on patient's core temperature?)

- If no, Prompt: who does set the temperature? What temperature is the machine usually set at?
- Under what circumstances would the temperature of the dialysate need to be adjusted from the prescribed temperature?
    - Does this change need approval from the caring physician? i.e. can you change the temperature as needed or do you need to consult with a physician in order to do this? When would you consult with a Dr.? Is it easy to consult with a Dr?

NOTE: if they do not already do this, ask in hypothetical ('would')

5. Can you describe the steps (that would be) involved in a typical clinical situation where you would set an individualized cooler dialysate temperature for a patient?
    - Prompt: who needs to do what before you can/could set individualized cooler dialysate temperatures for your patients?
    - Prompt (if not hypothetical): How often do you do this (for how many patients in a given day/week)?
    - Prompt: Why did/why would you set an individualized cooler dialysate temperature for that/a patient?
    - Prompt: When would you/do you do it?
    - Prompt: How do you keep track?

Social/Professional Role and Identity

6. Do/would you see it as your job/responsibility to set individualized cooler dialysate temperatures ?
    - Prompt: Is it anyone else's job/whose should it be? Should anyone else be involved?
        - Why? Who? Other health care professionals? Patients etc? Why? How should they be involved?

Intentions

7. Would you consider setting individualized cooler dialysate temperature for all the patients under your care? Why/Why not?
    - Prompt: In what situations may you find yourself *more* motivated to set the prescribed individualized cooler dialysate temperature? Why?
    - Prompt: In what situations may you find yourself *less* motivated to set the prescribed individualized cooler dialysate temperature? Why?

Beliefs about capabilities

8. Would you find it easy to set individualized cooler dialysate temperatures to all your patients? Why/why not?
    a. Can you give me some examples of anything that you think makes it (would make it) easy for you to set individualized cooler dialysate temperatures for all of your patients?

- Prompt: (give examples if needed and then ask them to elaborate: patient factors, colleagues, process of care, equipment used to take and set temperature, the unit etc.)

9. On the flipside, would you find it difficult to set individualized cooler dialysate temperatures for all of your patients? (Why? Under what circumstances wouldn't you?)
   a. Can you give me some examples of anything that you think may be challenging to set individualized cooler dialysate temperatures for all your patients?
   - Prompt: (e.g. patient factors, colleagues, process of care, equipment used to take and set temperature, the unit etc.)
   - Prompt: in what situations might you find yourself hesitant to set an individualized dialysate temperature for a patient?
     - Prompt: what would help put your mind at ease? Why are you hesitant in those situations?

Environmental context and resources

10. Do you foresee any logistical problems for setting individualized cooler dialysate temperatures for all the patients under your care?
11. Is there anything about the environment of the unit/ward itself that (could) influence whether or not you set individualized cooler dialysate temperatures?
    - Prompt: physical set-up/environment
12. What resources would be helpful/are needed in order for you to be able to set individualized cooler dialysate temperatures for all of your patients?
    a. To what extent are these resources available in your setting/center? (and how does this impact (or will it impact) your ability to set individualized cooler dialysate temperatures for the patients under your care?)

Knowledge

13. Are there any/do you use any guideline recommendations for individualizing cooler dialysate temperatures?
    - What are your local policies for setting dialysate temperatures in general?
    - Prompt: What are the guidelines for normal dialysis treatment? Would setting individualized cooler dialysis temperatures conflict with any guidelines or **local processes** currently used?
14. Have you come across any evidence linking dialysate temperature and patient symptoms or health outcomes?
    - Prompt: what is the evidence? What are your thoughts on this evidence, do you agree with it? Why or why not?
15. What information or knowledge would you need to be informed about promoting (in order to promote) individualized cooler dialysate temperature to all your patients?
    - Prompt: What would convince you? What information or evidence do you feel is lacking in order for you to promote individualized cooler dialysate temperatures to all your patients?

Beliefs about consequences

16. What are some of the benefits of setting individualized cooler dialysate temperature? (Prompts: for patients, yourself as a nurse, for the unit/ward?)
17. What are some of the negative aspects of setting individualized cooler dialysate temperature? (Prompts: for patients, yourself as a nurse, for the unit/ward?)
    - Prompt: I imagine some patients may describe feeling cold during dialysis treatment, do you do anything to help alleviate their symptoms?
        - Prompt: Do you offer any blankets? Warm tea? Etc. Prompt: Do you give patients any specific instructions to help relieve the cold symptoms they may experience during their dialysis treatment?Prompt: what are these instructions? E.g. Bring extra layers, bring blankets, etc.
    - Prompt: Do/would patients' emotions about cold symptoms affect your decision to set the individualized cooler dialysate temperature?
18. How does (how do you think) setting individualized cooler dialysate temperatures (will) influence your workload or capacity to do your job?
    a. If the patient is prescribed an individualized cooler dialysate temperature, how will this affect your practice? (i.e. a lot, a little? Why?)

Memory, attention and decision processes

19. Can you give me an idea of the kinds of situations that could come up where it may be easier to forget to set an individualized cooler dialysate temperature? What might help to avoid this?

- Prompt: and how can this be avoided?

Social influences

20. Who out of your colleagues also sets individualized cooler dyalisate temperatures (or are you the only person who would?) How does that impact on whether or not you do (would)?
21. Whose views impact most on whether or not you set individualised cooler dyalisate temperatures? Why?

Emotion

22. This may be a bit of an odd question, but bear with me, I have a psychology background! How do you think emotions, or tense situations on the unit/ward (will) influence you setting individualized cooler dialysate temperatures?
23. How comfortable are you about setting the dialysate temperature 0.5^{o}C below the patient's core temperature?
24. Can you describe to me the sorts of emotions you might have when (if) you set the cooler temperature? (e.g. guilt, worry, concern, satisfaction)

a. Do/would you ever feel worried or concerned about the cooler setting? (why/why not? What about the cooler setting makes you nervous?)
    - Prompt: Do/would these worries or concerns affect your decision to set the individualized cooler dialysate temperature? (How do these worries or concerns affect your decision? Why – or why not)

Goals

25. How much of a priority is setting individualized cooler dialysate temperatures in the grand scheme of everything else you do to reduce patients' symptoms?
    - Prompt: why is/isn't it a priority for you, is it something you would do in the future? Why/Why not?

Optimism

26. Overall, how optimistic are you that setting individual dialysate temperature will be good for your patients?

    - Prompt: Why? Does this influence whether or not you set a patient's individualized cooler dialysate temperature? Why/Why not? How?

Reinforcement

27. Are there any rewarding experiences that will encourage you to set individualized cooler dialysate temperatures? (e.g. satisfaction knowing that you're using evidence-based practice, doing everything you can to ensure good patient outcomes, rewarding part of job?) What rewards are in place that would help you set individualized cooler dialysate temperatures for the patients under your care?

Behaviour regulation

28. What do you think is needed to ensure that the correct individualized cooler dialysate temperature is consistently set for all patients?
    - Prompt: pre-programmed card that requests patient core temperature
29. Based on your experiences, do you have any suggestions or strategies that you would recommend for how we could go about implementing the setting of individualized cooler dialysate temperatures for all patients?
30. Last question!: Thinking about everything we've just discussed, what are the most important factors that would influence you setting individualized cooler dialysate temperatures for all your patients?

Is there anything else you'd like to say or expand on?

Thanks very much for your time.
