# Supplementary material for: Barriers and facilitators to healthcare professional behaviour change in clinical trials using the Theoretical Domains Framework: a case study of a trial of individualized temperature-reduced haemodialysis
Source: Trials. 2017 May 22;18:227. doi: 10.1186/s13063-017-1965-9 (PMC5440991; doi:10.1186/s13063-017-1965-9)
Supplement: Supplementary file 2 — Interview guide physicians. (PDF 98 kb) [file 13063_2017_1965_MOESM2_ESM.pdf]

Interview Guide - Physicians

Explanation:

- Thank you for agreeing to speak with me about using cooler dialysate temperatures
- Approximately 30-45 minutes – audio-recorded
- Free to not answer, leave at any time
- Anonymised transcripts
- May be overlap, some answers repeated ok
- Your input as a clinician on the dialysis unit is very useful as we're trying to determine the best way to do a study on the effects of cooler dialysate temperatures on patient outcomes. So you know, my background is in psychology and I'm not a clinician. I'm interested in your views, experience and insight into the use of cooler dialysis temperatures. There are no right or wrong answers, and I'll probably ask you for clarification throughout our discussion.
- Any questions before we start?

Background of Informant

- Male or female (to keep track of, won't be asked)
- Confirm if right campus documented

1. What is your job title?
2. How long have you been a (job title)?

All of the following questions will concern the use of cooler dialysate temperatures. In relation to that, I would like you to think of:

You personally ordering/prescribing individualized cooler dialysate temperatures for every patient in the next month.

When we say "individualized cooler dialysate temperature" we mean the temperature of the dialysate set 0.5^{o}C below the patient's core temperature.

If at any point you'd like this definition repeated, please let me know.

To start, I'd like to get an idea of your usual procedure for ordering/prescribing dialysate temperatures, and whether ordering/prescribing individualized cooler dialysate temperatures for your patients is something that you ever do?

3. What temperature of dialysate do you usually/would you prescribe/order for your patients (e.g. a static and specific temperature [36.5 ^{o} C] or a variable temperature dependent on the patient's core temperature at dialysis?) Why?

NOTE: if they do not already do this, ask in hypothetical ('would')

4. Can you describe the steps (that would be) involved in a typical clinical situation where you prescribe/order an individualized cooler dialysate temperature for a patient?
    - Prompt (if not hypothetical): How often do you do this (for how many patients in a given day/week)?
    - Prompt: Why did/why would you prescribe/order an individualized cooler dialysate temperature for that/a patient?
5. When/if you were to prescribe/order an individualized cooler dialysate temperature for a patient, when do/would you do it? (e.g. before the patient's first dialysis session; after the first week of dialysis, etc.)
    a. How do/would you keep track of whether you have prescribed/ordered an individualized dialysate temperature?
    b. If you do/were to prescribe/order an individualized cooler dialysate temperature for a patient, how would you let the personnel on the unit know this? How does this decision (this prescription) get communicated to the unit staff?

Intentions

6. Would you consider prescribing individualized cooler dialysate temperatures for all of your patients? Why/Why not?
    - Prompt: Would you prescribe individualized cooler dialysate temperatures for your patients in the future? Why/why not?
    - Prompt: how motivated are you to do this? Why/Why not?

Beliefs about capabilities

7. Would you find it easy to prescribe/order individualized cooler dialysate temperatures for all your patients? Why/Why not?
    a. Can you give me some examples of anything that you think makes it/would make it easy to prescribe/order individualized cooler dialysate temperatures for all your patients?
        - Prompt: (e.g. patient factors, colleagues, process of care, equipment, the unit etc.)
        - Prompt: how does (the facilitator) make it (would make it) easier to prescribe/order individual dialysate temperatures? Why?

8. Now on the flipside, how difficult would it be to prescribe/order individualized cooler dialysate temperatures to all your patients? (Why? For which patients wouldn't you? Under what circumstances wouldn't you? Why?)
    a. Can you give me some examples of anything that you think may be challenging or that would make it less likely that you would prescribe/order individualized cooler dialysate temperatures for all your patients?
    - Prompt: (e.g. patient factors, colleagues, process of care, equipment, the unit etc.)
    - Prompt: in what situations may you find yourself hesitant to order individualized cooler dialysate temperatures for your patients? Why? What would help put your mind at ease?

9. How confident are you/would you be about personally prescribing/ordering individualized cooler dialysate temperatures for <u>all</u> your patients?
    - Prompt: Why/why not, what factors make you less confident? What factors make you feel more confident that you can?
        a. And which of these factors do you feel would most influence you when prescribing individualized cooler dialysate temperatures? (clarify if the influence is positive or negative if not clear)

Environmental Context and Resources

10. Do you foresee any logistical problems for prescribing/ordering individualized cooler dialysate temperatures for all patients?
11. Is there anything about the environment of the unit/ward itself that (could) influence whether or not you prescribe individualized cooler dialysate temperatures?
    - Prompt: physical environment
12. What resources would be helpful/are needed in order for you to be able to prescribe/order individualized cooler dialysate temperatures for all of your patients?
        a. To what extent are these resources available in your setting/centre? (and how does this impact (or will it impact) your ability to prescribe/order individualized cooler dialysate temperatures to all your patients?)
    - Prompt: based on the barriers and facilitators discussed earlier

Knowledge

13. Are there any/do you use any guideline recommendations for prescribing individualized cooler dialysate temperatures?
        - What are your local policies for prescribing dialysate temperatures in general?
        - Prompt: what are the guidelines for prescribing dialysate temperatures normally? Would prescribing individualized cooler dialysate temperatures conflict with any **local processes** of guidelines that you currently use?
14. Have you come across any evidence linking dialysate temperature and patient symptoms or health outcomes?
        - Prompt: what is the evidence? What are your thoughts in this evidence, do you agree with it? Why or why not?
15. What information or knowledge would you need to be informed about for prescribing/ordering individualized cooler dialysate temperatures for all your patients?
        - Prompt: What would convince you? What information or evidence do you feel is lacking in order for you to prescribe/order individualized cooler dialysate temperatures for all your patients?

Beliefs about consequences

16. What are some of the benefits of prescribing/ordering individualized cooler dialysate temperatures?
    - Prompts: benefits to patients (better health outcomes), yourself as a physician, for the unit/ward?
17. What are some of the negative aspects of prescribing/ordering individualized cooler dialysate temperatures? Would these influence whether you do this?
    - Prompts: to patients (symptoms), yourself as a physician/for the unit/ward?
    - Since we're asking about patient symptoms here, ask about any instructions they give about feeling cold, do patient's descriptions of feeling cold influence whether or not they order an individualized cooler temperature etc. – Patient symptoms come up a lot I want to try to only ask about them once or twice, so it doesn't get too redundant
18. How does (how do you think) prescribing/ordering individualized cooler dialysate temperatures (will) influence your workload or capacity to do your job?

Memory, Attention and Decision Processes

19. Can you give me an idea of the kinds of situations that could come up where it may be more likely to forget to prescribe/order an individualized cooler dialysate temperature? (if any mentioned, then ask: what might you find helpful to minimise this?)
20. Can you tell me about any competing demands that may interfere with you prescribing individualized cooler dialysate temperatures for all your patients?
    - Prompt: what are these demands? What would help you manage these multiple demands i.e. physical or resource factors?
    - On the flipside, is there anything that you ***do*** that (would) sets the stage or makes it easier to?
21. Thinking of your usual workday, to what extent do you think you can/or will be able to focus down on ordering/prescribing individualized cooler dialysate temperatures for all your patients?

Social/Professional Role and Identity

22. Do/would you see it as your job to prescribe/order individualized cooler dialysate temperatures?
    - Prompt: Is it anyone else's job/whose should it be? Should anyone else be involved in the decision regarding the prescription/ordering of individualized cooler dialysate temperatures?
        - Why? Who? Other health care professionals? Patients etc? Why? How should they be involved?

Social Influences

23. Can/could any of your colleagues working on the unit also prescribe/order individualized cooler dialysate temperatures for your patients? Or are you the only person who could?

24. Whose views impact most on whether or not you prescribe/order individualized cooler dialysate temperatures? Why?
    - Prompt: other physicians, nurses, patients?

Emotion

25. How do you think emotions, or tense situations on the unit/ward (will) influence you prescribing/ordering individualized cooler dialysate temperatures?
26. How comfortable are you about ordering dialysate temperature 0.5 ^{o}C below the patient's core temperature?
    - Prompt: Can you describe to me the sorts of emotions you might have when you (if you) prescribe/ order the cooler dialysate temperatures? (e.g. guilt, worry, concern, satisfaction)
        a. Do/would you ever feel worried or concerned about the cooler temperature?
    - Prompt: Do/would these worries or concerns affect you when prescribing/ordering individualized cooler dialysate temperatures? Why/Why not? How?

Goals

27. How much of a priority is prescribing individualized cooler dialysate temperatures in the grand scheme of everything you do to reduce patient symptoms?
    - Prompt: why is/isn't it a priority for you? Why/Why not?

Optimism

28. Overall, how optimistic are you that ordering/prescribing individualized cooler dialysate temperatures will be good for your patients?
    - Prompt: Why? Does this influence whether or not you prescribe/order individualized cooler dialysate temperatures for your patients? Why/Why not? How?

Reinforcement

29. Are there any rewarding experiences that will encourage you to prescribe/order individualized cooler dialysate temperatures for all of your patients? (e.g. satisfaction knowing that you're using evidence-based practice, doing everything you can to ensure good patient outcomes, rewarding part of your job?) What rewards are in place that would help you prescribe/order individualized cooler dialysate temperatures to all of your patients?

Behavioural Regulation

30. What do you think is needed to ensure that individualized cooler dialysate temperatures are prescribed/ordered for all patients?
31. Based on your experiences, do you have any suggestions, thoughts or strategies you would recommend for how we should go about implementing the prescription/ordering of

individualized cooler dialysate temperatures for all patients? If this were to be implemented on your unit, how could be best help you?

32. Final question!: Thinking about everything we've just discussed, in your opinion, what are the most important factors that would influence whether you prescribe/order individualized cooler dialysate temperatures for all your patients?

Is there anything else you'd like to say or expand on?

Thanks very much for your time.
