# Supplementary material for: Barriers and facilitators to healthcare professional behaviour change in clinical trials using the Theoretical Domains Framework: a case study of a trial of individualized temperature-reduced haemodialysis
Source: Trials. 2017 May 22;18:227. doi: 10.1186/s13063-017-1965-9 (PMC5440991; doi:10.1186/s13063-017-1965-9)
Supplement: Supplementary file 3 — Final coding manual. (PDF 244 kb) [file 13063_2017_1965_MOESM3_ESM.pdf]

<u>MyTEMP Data Analysis Codebook</u>

TACT-A Specified Behaviours

** Note: behaviour refers to individualized* ***and/or*** *cooler dialysate temperature **

Physicians (A- actor)

Action: Prescribe (or make a standing order for/use a unit wide policy/medical directive for) individualized cooler dialysate temperatures where each patient's dialysate temperature is set 0.5ºC cooler than the patient's core temperature

a) Inputted directly by the physician into a software program or;
b) Prescriptions are made by a physician (either on paper or in electronic form) and then inputted into the necessary software by a nurse

Target: Nurses

Time: Before roll-out of the study

Context: The Dialysis Unit (or office, wherever the physician has access to the program to input orders)

Nurses (A – actor)

Actions: Setting the dialysate temperature to 0.5 degrees below patient's core body temperature; which involves:

- Take core body temperature of each patient at the beginning of each treatment session using the usual method (tympanic/oral)
- Set temperature of the dialysate to 0.5ºC below the patient's core body temperature (subtract 0.5ºC from the measured core body temperature)
- Input that value into the dialysis machine manually as the dialysate temperature for each treatment session

Target: Each patient undergoing dialysis

Time: At the beginning of dialysis treatment ("run up" part of set up of machine before connecting patient)

Context: In the dialysis unit, at the dialysis machine at the patient's bedside

Coding instructions:

1. The framework used for data analysis is a combination of the 12-Domain Theoretical Domains Framework (TDF1; Michie et al., 2005) and the 14-Domain TDF2 (Cane et al., 2012) with one additional theme relevant to broader trial implementation but not specific to the TACT-specified behaviour of interest; note: this may eventually be an emergent theme(s).
2. Code into domains, and wherever possible use its corresponding constructs to justify coding the text into a domain (Definitions of constructs and domains are included below). Note to double coder: the double coding is at the domain level, but use constructs to inform the decision of which domain to code.
3. Code all responses (both positive and negative, clarifying the directionality of the quote wherever possible, using verbatim quotes if possible).
4. Code all relevant text into each domain (i.e. provide context to demonstrate how the text is associated with a domain), but also highlight the specific sections of the text within that are specifically related to the domain**. Note to double coder: only code the highlighted text, but use the broader context text to inform the coding. Can code un-highlighted text if deemed relevant.**
5. Please use the "Decision Rule" columns to supplement the description of the domains and constructs for this context. Also see the decision rule column for information regarding text that could be justifiably coded into multiple domains. Can code highlighted text to multiple domains if necessary.
6. Note that definitions are from Cane et al. (2012) unless otherwise indicated. Wherever possible, use the definitions provided in this codebook to justify coding
7. Code "No" answers along with the corresponding question into the appropriate domains.

**Domain**	Construct	Decision Rule	Example
**1. Knowledge**<br>*An awareness of the existence of something*<br><br>*What do they know and how does that influence what they do? (AP/JP)*	Knowledge (including knowledge of condition/scientific rationale): *An awareness of the existence of something*<br>Procedural knowledge: *Knowing how to do something*<br>Knowledge of task environment: *Knowledge of the social and material context in which a task is undertaken.*	<u>Consider coding to this domain:</u><br>Discussion about evidence related to the use of cooler dialysate temperatures (or lack thereof)<br><br>Awareness of guidelines, or lack of guidelines (if describe guidelines or unit policies, also code at Goals, particularly if numerical values of standard temperatures on unit described)<br><br>Description of conflicts between unit policy/guidelines and individualized cooler dialysate temperature (Also code this information at Goals)<br><br>Anecdotal evidence regarding the use of individualized and/or cooler dialysate temperatures<br><br>Descriptions of evidence that would convince	"Now maybe I need to review the evidence and see what's out there but **I'm pretty sure there's not a lot of long-term impact or good quality evidence on the impact of lower dialysate temperature in terms of patient outcomes**." (Physician#1)

		them to use cooler dialysate temperatures<br><br>Procedural knowledge: Tends to be hypothetical – knowing how the process of prescribing/setting individualized cooler dialysate temperatures would be done.<br><br>Inappropriate coding to this domain:<br>Discussion of who sets/prescribes cooler temperatures (Social Professional Role and ID), discussion of how easy/difficult setting/prescribing cooler dialysate temperatures is/will be (Beliefs About Capabilities)	
**2. Skills**<br>*An ability or proficiency acquired through practice*	Skills: *An ability or proficiency acquired through training and/or practice*<br><br>Skills development: *The gradual acquisition or advancement through progressive stages of an ability or proficiency acquired through training and practice*<br><br>Competence: *One's repertoire of skills, and ability especially as it is applied to a task or set of tasks*<br><br>Ability: *Competence or capacity to perform a physical or mental act. Ability may be either unlearned or acquired by education and practice*<br><br>Interpersonal skills: *An aptitude enabling a person to carry on effective relationships with others, such as an ability to cooperate, to assume appropriate social responsibilities or to exhibit adequate flexibility*<br><br>Practice: *Repetition of an act, behaviour, or series of activities, often to improve performance or acquire a skill*<br><br>Skills assessment: *A judgement of the quality, worth, importance. Level or value of an ability or proficiency acquired through training and practice*	Consider coding to this domain:<br><br>Discussion of how the relationship/rapport between health care professional and patient may facilitate the use of individualized cooler dialysate temperatures (Interpersonal Skills)<br><br>Inappropriate coding to this domain: What they normally do currently – code to 'Nature of the Behaviour' instead.<br><br>Discussion of communication strategies already used with patients when discussing aspects of treatment, or changes in treatment - code to 'Behaviour Regulation' instead.	"Respondent: That's right, yes. Yes and it's their treatment too. You have to make sure that you inform them. It's just probably how well the patient knows you.<br><br>Interviewer: Okay, so the relationship between…?<br><br>Respondent: Yes, that's right. **Yes, the rapport that you have with the patient as well. "** (Nurse#1)

**3. Social/Professional Role and ID**<br>*A coherent set of behaviours and displayed personal qualities of an individual in a social or work setting*<br><br>*How does who they are as a HCP influence whether they do something or not? (AP/JP)*	Professional identity: *The characteristics by which an individual is recognised relating to, connected with or befitting a particular profession*	<u>Consider coding to this domain:</u> discussion about who prescribes/sets cooler temperature. Specifically what each different health care provider does as it relates to (or would relate to) setting/prescribing individualized cooler dialysate temperatures to all patients.<br><br>What participants usually do when prescribing/ setting cooler dialysate temperatures. (Also coded at Nature of Behaviour)<br><br>Descriptions of 'shared care' models<br><br>More specific than social influence. A description of what someone else is doing (e.g., "Physicians prescribe" "Nurses set")<br><br>Descriptions of how some nurses can modify dialysate temperatures during treatment<br><br><u>Inappropriate coding to this domain:</u> If participant describes relationship i.e. a need for someone else's behaviour, code at 'Social Influences' instead.	"What we normally do is **the doctor normally orders the hemodialysis order** to 36.5. That's for everybody, all the patients. **However, our nurses can change the temperature** whenever the blood pressure of the patient is lower." (Nurse#1)
	Professional role: *The behaviour considered appropriate for a particular kind of work or social position*		
	Social identity: *The set of behavioural or personal characteristics by which an individual is recognizable [and portrays] as a member of a social group*		
	Identity: *An individual's sense of self defined by a) a set of physical and psychological characteristics that is not wholly shared with any other person and b) a range of social and interpersonal affiliations (e.g., ethnicity) and social roles.*		
	Professional boundaries: *The bounds or limits relating to, or connected with a particular profession or calling*		
	Professional confidence: *an individual's belief in his or her repertoire of skills and ability especially as it is applied to a task or set of tasks.*		
	Group identity: *the set of behavioural or personal characteristics by which an individual is recognizable [and portrays] as a member of a group*		
	Leadership: *The processes involved in leading others, including organising, directing, coordinating and motivating their efforts toward achievement of certain group or organization goals*		
	Organizational commitment: *An employee's dedication to an organisation and wish to remain part of it. Organisational commitment is often described as having both an emotional or moral element and a more prudent element*		

**4. Beliefs About Capabilities**<br>*Acceptance of the truth, reality, or validity about an ability, talent or facility that a person can put to constructive use*<br><br>*Do they think they can do what they should so and how does that influence whether they do it or not? (AP/JP)*	Self-confidence: *Self-assurance or trust in one's own abilities, capabilities and judgement*<br>Perceived competence: *An individual's belief in her or her ability to learn and execute skills*<br>Self-efficacy: *An individual's capacity to act effectively to bring about desired results, as perceived by the individual*<br>Perceived behavioural control: *an individual's perception of the ease or difficulty of performing the behaviour of interest*<br>Beliefs: *The thing believed; the proposition or set of propositions held true*<br>Self-esteem: *The degree to which the qualities and characteristics contained in one's self-concept are perceived to be positive*<br>Empowerment: *The promotion of the skills, knowledge and confidence necessary to take great control of one's life as in certain educational or social schemes; the delegation of increase decision-making powers to individuals or groups in a society or organization*<br>Professional confidence: *An individual's beliefs in his or her repertoire of skills, and ability, especially as it is applied to a task or set of tasks.*	Consider coding to this domain:<br>Descriptions of how easy or difficult it will/would be to do the behaviour (Physicians – prescribing cooler dialysate temperatures; Nurses/Technicians – setting cooler dialysate temperatures)<br><br>Descriptions of how confident a participant feels that they would be able to set or prescribe an ICDT	"**It's easy** and I can basically just do it [prescribe a cooler dialysate temperature] and it'll get done." (Physician#1)
**5. Optimism**<br>*The confidence that things will happen for the best or that desired goals will be attained*	Optimism: *The attitude that outcomes will be positive and that people's wishes or aims will be ultimately fulfilled*<br>Pessimism: *The attitude that things will go wrong and that people's wishes or aims are unlikely to be fulfilled*<br>Unrealistic optimism: *the inert tendency for humans to over-rate their own abilities and chances of positive outcomes compared to those of other people*	Consider coding to this domain:<br><br>Participants' descriptions of their level of optimism regarding the effectiveness of individualized cooler dialysate temperatures on patient outcomes. (May be hypothetical – optimistic for study)<br><br>Code both positive and negative answers	

**6. Beliefs about Consequences**<br>*Acceptance of the truth, reality or validity about outcomes of a behaviour in a given situation*	Beliefs: *The thing believed; the proposition or set of propositions held true*<br><br>Outcome expectancies: *Cognitive, emotional, behavioural, and affective outcomes that are assumed to be associated with future or intended behaviour. These assumed outcomes can either promote or inhibit future behaviours.*<br><br>Characteristics of outcome expectancies: *Characteristics of the cognitive, emotional and behavioural outcomes that individuals believe are associated with future or intended behaviours and that are believed to either promote or inhibit these behaviours. These include whether they are sanctions/rewards, proximal/distal, valued/not valued, probable/improbable. Salient/not salient, perceived risks or threat*s.<br><br>Anticipated regret: *A sense of the potential negative consequences of a decision that influences the choice made: for example an individual may decide not to make an investment because of the feelings associated with an imagined loss*<br><br>Consequents: *An outcome behaviour in a given situation*	Consider coding to this domain:<br><br>Positive and negative outcomes for prescribing or setting individualized cooler dialysate temperatures (includes patient symptoms, impact on health care professionals, and impact on unit).<br><br>Beliefs about treatment outcomes – both theoretical and based on experience due to using individualized **and/or** cooler dialysate temperatures<br><br>Descriptions/explanations of how cooler dialysate temperatures are beneficial<br><br>Potential long term outcomes of using individualized **and/or** cooler dialysate temperatures<br><br>Descriptions of clinical factors that influence the prescribing or setting of individualized and/or cooler dialysate temperatures (Also code at 'Environmental Context and Resources')<br><br>Note: clinical factors include fever, hypo/hypertension, cardiac issues, feeling cold etc. as well as descriptions of patients doing well on dialysis i.e. have a lack of clinical features<br><br>Impact on workload<br>Health care professionals talking about other health care professionals (e.g. a physician discussing how nurses' workload will be impacted by the use of individualized cooler dialysate temperatures)<br><br>Descriptions of concerns regarding thermometer accuracy (also code at 'Environmental Context and Resources')	E.g. using cool dialysate temperatures helps manage hypotension/fluid removal during dialysis<br><br>"I think the main benefit is preventing or avoiding intradialytic hypotension" (Physician#1)<br><br>Clinical factors= fever, hypo/hypertension, cardiac issues etc.

		Describing learning curves, or getting in the habit of setting or prescribing ICDTs.<br><br><u>Inappropriate coding to this domain:</u><br>Descriptions of patients feeling cold (can code to social influences when patient emotion/distress/or complaints about being cold are described)	
**7. Reinforcement**<br>*Increasing the probability of a response by arranging a dependent relationship, or contingency, between the response and a given stimulus*<br><br>*How have their experiences (good and bad) of doing it in the past influence whether or not they do it? (AP/JP)*	Rewards (proximal/distal, valued/ not valued, probable/improbable): *Return or recompense made to, or received by a person contingent on some performance.*<br><br>Incentives: *An external stimulus, such as condition or object, that enhances or serves as a motive for behaviour*<br><br>Punishment: *The process in which the relationship between as response and some stimulus or circumstance results in the response becoming less probable; a painful, unwanted or undesired event or circumstance imposed as a penalty on a wrongdoer*<br><br>Consequents: *An outcome of behaviour in a given situation*<br><br>Reinforcement: *A process in which the frequency of a response is increased by a dependent relationship or contingency with a stimulus*<br><br>Contingencies: *A conditional probabilistic relation between two events. Contingencies may be arranged via dependencies or they may emerge by accident*<br><br>Sanctions: *A punishment or other coercive measure, usually administered by a recognized authority, that is used to penalise and deter inappropriate or unauthorized actions.*	<u>Consider coding to this domain:</u><br><br>Reinforcement/reward of setting/prescribing cooler dialysate temperatures based on past experience.<br>Consider social reinforcement punishment, as well as intrinsic reward<br><br>Also code 'no' answers<br><br>Can also include hypothetical reinforcement/reward	e.g. patient and nurse satisfaction with treatment: "Oh, yes because the patients are very satisfied when they say, "Oh…" Like last treatment I was unable to remove a lot of fluids but this treatment I was and there's no complication. As long as they're comfortable, no complications during dialysis and **I'm able to make them comfortable after and make them happy. That's rewarding.**" (Nurse#1)

**8. Intention**<br>*A conscious decision to perform a behaviour or a resolve to act in a certain way*<br><br>*How does how inclined they are to do something influence whether they will do it? (AP/JP)*	Stability of intentions: *ability of one's resolve to remain in spite of disturbing influences*<br>Stages of Change model: *A model that proposes that behaviour change is accomplished through five specific stages*<br>Transtheoretical model and stages of change: *a five-stage theory to explain changes in people's health behaviour. It suggests that change takes time, that different interventions are effective at different stages, and that there are multiple outcomes occurring across the stages*	<u>Consider coding to this domain:</u> Participant's descriptions of how motivated they are to prescribe or set individualized and/or cooler dialysate temperatures<br><br>Participant's inclinations to prescribe/set individualized cooler dialysate temperatures to all patients.<br><br>Participants' descriptions of when they are and are not inclined to use *cooler* dialysate temperatures. Also code to other domains as necessary (i.e. 'Beliefs about Consequences')<br>**Note:** Indicator of intention must be explicit and not inferred<br><br>Participants' descriptions of how inclined they are to use *individualized* cooler dialysate temperatures. Also code to other domains as necessary (i.e. 'Beliefs about Consequences')<br><br>Importance of staff buy-in<br><br><u>Inappropriate coding to this domain:</u> this is different from how effective they think using individualized and/or cooler dialysate temperatures will be or that cooler dialysate temperatures help manage hypotension and help with fluid removal during treatment ('Beliefs About Consequences') and different from how much of a priority prescribing/setting individualized and/or cooler dialysate temperatures is for them ('Goals').<br>Be careful not to code the reasons for the intention (focus on statements that directly reflect their intention and motivation)	"having the core temperature as your baseline and as your guide really ensures that everybody's going to get a bit of cooler dialysate rather than something a bit more random. **I personally would love to do it**." (Physician#1)<br><br>Example of intention:<br>**"if they're doing well and there's no issues there why bother.**" (Physician#1)<br>Not intention (coded at Beliefs about consequences and Environment): "I use it in situations where the blood pressures are low or the patient has a tendency towards lower blood pressures**"** (Physician#13)

**9. Goals** *Mental representations of outcomes or end states that an individual wants to achieve*<br><br>*How important is what they do and does that influence whether or not they do it? What standards are they trying to reach, how does that influence whether or not they do it? (AP/JP)*	Goals (distal/proximal): *Desired state of affairs of a person or system, these may be closer (proximal) or further away (distal)*<br>Goal priority: *Order of importance or urgency of end state toward which one is striving*<br>Goal/target setting: *A process that establishes specific time based behavioural targets that are measureable, achievable and realistic*<br>Goals (autonomous/controlled): *The end state toward which one is striving: the purpose of an activity or endeavour. It can be identified by observing that a person ceases or changes their behaviour upon attaining this state; proficiency in a task to be achieved within a set period of time.*<br>Action planning: *The action or process of forming a plan regarding a thing to be done or a deed*<br>Implementation intention: *The plan that one creates in advance of when, where an how one will enact a behaviour*	<u>Consider coding to this domain:</u><br><br>Descriptions of whether or not prescribing/setting ICDTs is a priority<br><br>Unit standards for dialysate temperature: e.g. 36.5, 36<br><br>Descriptions of how setting/prescribing individualized cooler dialysate temperatures are (or are not) in conflict with guidelines/local policies currently used (also code this at 'Knowledge') or in conflict with other aspects of the care they provide (goal conflict)<br><br>Unit standards (double coded at knowledge)	"Interviewer:<br><br>How much of a priority is prescribing individualized cooler dialysate temperatures in the grand scheme of everything you do?<br><br>Respondent: Very low" (Physician#1)
**10. Memory, Attention and Decision Processes** *The ability to retain information, focus selectively on aspects of the environment and choose between two or more alternatives*	Memory: *The ability to retain information or a representation of a past experience, based on the mental processes of learning or encoding retention across some interval of time, and retrieval or reactivation of the memory; specific information of a specific task*<br>Attention: *A state of awareness in which the senses are focussed selectively on aspects of the environment and the central nervous system is in a state of readiness to respond to stimuli*<br>Attention control: *The extent to which a person can concentrate on relevant cues and ignore all irrelevant cues in a given situation*<br>Decision making: *The cognitive process of choosing between two or more alternatives, ranging from the relatively clear-cut to the complex*<br>Cognitive overload/tiredness: *The situation in which the demands placed on a person by mental work are greater than a person's mental abilities*	<u>Consider coding to this domain:</u><br><br>When/why would it be easy to forget.<br><br>Descriptions of decision processes regarding dialysate temperature (reasons why dialysate temperature chosen)<br><br>Due the hypothetical nature of the interviews, please also code participant's descriptions of when they think they would forget as well as reasons why they don't think they would forget at this domain	"Interviewer:<br>Okay. Are there certain types of situations that could come up where it may be more likely to forget?<br><br>Respondent:<br>A busy day, or a bad day – if the patients are having a bad day. Shortage, staffing shortage, you know?" (Nurse#10)<br><br>"In a sense it's fairly easy to be swayed that if you're going to choose why not choose the one that may be helpful and certainly is not harmful." (Physician#1)

**11. Environmental Context and Resources**<br>*Any circumstance of a person's situation or environment that discourages or encourages the development of skills and abilities, independence, social competence, and adaptive behaviour*<br><br>*What are the things in their environment that influence what they do and how do they influence? (AP/JP)*	Environmental stressors*: External factors in the environment that cause stress*<br>Resources/material resources: *Commodities and human resources used in enacting a behaviour*<br>Organizational culture/climate: *A distinctive pattern of thought and behaviour shared by members of the same organization and reflected in their language, values, attitudes, beliefs and customs*<br>Salient events/critical incidents: *Occurrences that one judges to be distinctive, prominent or otherwise significant*<br>Person x environment interaction: *Interplay between the individual and their surroundings*<br>Barriers and facilitators: *In psychological contexts, barriers/facilitators are mental, emotional or behavioural limitations/strengths in individuals or groups*	<u>Consider coding to this domain:</u><br><br>Descriptions of thermometer type<br><br>Description of dialysis machine type/added modules (e.g. blood temperature monitor)<br><br>Availability of resources to reduce patient symptoms of feeling cold (e.g. blankets; also code descriptions of using blankets for cold symptoms at 'Behaviour Regulation')<br><br>Discussion about limitations of dialysis machines as it relates to setting ICDTs (i.e. parameters for dialysate temperature setting)<br><br>Descriptions of software/EMR used<br><br>Context of the unit itself that would/does influence whether cooler dialysate temperatures are/would be set.<br><br>Descriptions of how more time will be required for setting/prescribing ICDT.<br><br>Descriptions of how environmental/contextual factors can influence the accuracy of temperature measure (Also code these descriptions at 'Beliefs about Consequences')<br><br>Patient clinical factors that would influence whether or not a cooler dialysate temperature was prescribed/set for a patient (Also code at 'Beliefs about Consequences')<br>Note: clinical factors include fever, hypo/hypertension, cardiac issues, feeling cold etc. as well as descriptions of patients doing well on dialysis (i.e. have a lack of clinical features) and patient comfort	e.g. equipment used to set/prescribe temperature, thermometers, blankets,<br>electronic recording "EMR" software used<br><br>"Well, so equipment might be a limitation depending on the patient's core temperature. The equipment has maximum and minimum setting of temperature." (Physician#1)<br><br>"However, with the temperature outside now and then when they come, what we normally do pre-dialysis is we want to settle them a little bit because it's cold outside. When they come in and we check their temperature, they're already cold because we normally check the ear temperature. Yes, tympanic temperature. Because **they come from outside and they never cover their ears, normally, it's low already**. " (Nurse#1)

**12.Social Influences**<br>*Those interpersonal processes that can cause individuals to change their thoughts, feelings, or behaviours*<br><br>*What do others think of what they do? Who are they and how does that influence what they do?*	Social pressure: *the exertion of influence on a person or group by another person or group*<br>Social norms: *Socially determined consensual standards that indicate a) what behaviours are considered typical in a given context and b) what behaviours are considered proper in the context*<br>Group conformity: *The act of consciously maintaining a certain degree of similarity to those in your general social circles*<br>Social comparisons: *The process by which people evaluate their attitudes, abilities or performance relative to others*<br>Group norms: *Any behaviour, belief, attitude or emotional reaction held to be correct or acceptable by a given group in society*<br>Social support: *The apperception or provision of assistance or comfort to others, typically in order to help them cope with a variety of biological, psychological and social stressors. Support may arise from any interpersonal relationship in an individual's social network, involving friends, neighbours, religious institutions, colleagues, caregivers of support groups*<br>Power: *The capacity to influence others, even when they try to resist this influence*<br>Intergroup conflict: *Disagreement or confrontation between two or more groups and their members. This may involve physical violence, interpersonal discord, or psychological tension.*<br>Alienation: *Estrangement from one's social group; a deep seated sense of dissatisfaction with one's personal experiences that can be a source of lack of trust in one's social or physical environment or in oneself; the experience of separation between thoughts and feelings*<br>Group identity: *the set of behavioural or personal characteristics by which an individual*	<u>Consider coding to this domain</u>:<br><br>Discussion about how others influence whether or not a cooler dialysate temperature is set/prescribed. Includes patient influence, and influence of other healthcare professionals.<br><br>Descriptions of patient's complaints related to feeling cold, resistance to having cooler dialysate temperatures set.<br><br>Discussing the importance of patient buy-in<br><br>Discussion of the need for other's activity "We need a doctor's order" (e.g., nurse describing how they would need a physician order)<br><br>Descriptions of how nurses follow doctor's orders<br><br>Recommending a medical directive/protocol so nurses can set ICDTs without requiring a doctor's order<br><br>For physicians/nurse practitioner/prescriber: descriptions of notifying nurses of changes in prescription in person or notes etc.<br><br>Descriptions of unit-wide pattern of thought related to cooler dialysate temperatures (considered social norms)<br><br><u>Inappropriate coding to this domain:</u><br>Specific descriptions of the roles of others i.e. what someone else is doing "Physicians prescribe" "Nurses set" should be coded at 'Social Professional Role and Identity.'	"R:They are, they are because sometimes mind over matter and they say, "Okay, set this up." **Then they disagree with you but they still set it up and then they'll say, "I'm really cold. I'm really cold. I'm really cold." They won't stop.**<br><br>I:Then in that case their temperature…?<br><br>R**:Yes, in that case you have to increase the temperature**, and show it to them, in the machine that, "Look, I did it." (Nurse#1)<br><br>"**I think everyone, most people in Ottawa do.** I'll be very honest. Most people use cooler than core temperature. Well assuming core temperature is 37, most people use cooler than core temperature." (Physician#1)

	is recognizable [and portrays] as a member of a group		
	Modelling: *In developmental psychology the process in which one or more individuals or other entities serve as examples (models) that a child will copy*		
**13. Emotion**<br>*A complex reaction pattern, involving experiential, behavioural and physiological elements, by which the individual attempts to deal with a personally significant matter or event*<br><br>*How do they feel about what they do and do those feelings influence what they do? (AP/JP)*	Fear: *An intense emotion aroused by the detection of imminent threat, involving an immediate alarm reaction that mobilizes the organism by triggering a set of physiological changes*	<u>Consider coding to this domain:</u><br><br>Descriptions of emotions experienced by nurses/nephrologists when setting/prescribing cooler and/or individualized cooler dialysate temperatures (can be positive or negative)<br><br>Descriptions of when participant (i.e. nephrologist, nurse) would be worried/concerned about the cooler dialysate temperatures<br><br>Include 'no' answers<br><br><u>Inappropriate coding to this domain:</u><br>Descriptions of patients' emotions regarding cooler dialysate temperatures (code at 'Social Influences' instead).	"I don't think so. I don't think **– I can't sense any resistance or strong feelings about temperature**." (Physician#1)
	Anxiety: *A mood state characterized by apprehension and somatic symptoms of tension in which an individual anticipates impending danger, catastrophe or misfortune.*		
	Affect: *An experience or feeling of emotion, ranging from suffering to elation, from the simplest to the most complex sensations of feelings, and from the most normal to the most pathological emotional reactions.*		
	Stress: *A state of physiological or psychological response to internal or external stressors*		
	Depression: *A mental state that presents with depressed mood, loss of interest or pleasure, feelings of guilt or low self-worth, disturbed sleep or appetite, low energy, and poor concentration*		
	Positive/negative affect: *the internal feeling/state that occurs when a goal has/has not been attained. A source of threat has/has not been avoided, or the individual is/is not satisfied with the present state of affairs*		
	Burn-out: *Physical, emotional or mental exhaustion, especially in one's job or career, accompanied by decreased motivation, lowered performance and negative attitudes towards oneself and others*		

**14. Behavioural Regulation**<br>*Anything aimed at managing or changing objectively measured actions*	Self-monitoring: *A method used in behavioural management in which individuals keep a record of their behaviour, especially in connection with efforts to changes or regulate the self; a personality trait reflecting an ability to modify one's behaviour in response to a situation*<br>Breaking habit: *to discontinue a behaviour or sequence of behaviours that is automatically activated by relevant situational cues*<br>Action planning: *The action or process of forming a plan regarding a thing to be done or a deed.*	<u>Consider coding to this domain:</u><br><br>Self-regulatory strategies already in place that would influence the prescription/setting of cooler dialysate temperatures. Focus on self-regulatory strategies only (not all strategies)<br><br>Coping plans, problem solving scripts/strategies used in response to patients' resistance to cooler dialysate temperatures (including providing blankets)<br><br>Descriptions of dialysate temperatures being increased in response to patients' complaints of being cold or in response to other clinical factors (e.g. hypertension)<br><br>Descriptions of auditing or spot-checks recommended for implementation<br><br><u>Inappropriate coding to this domain:</u> strategies suggested by the participant as being useful to implement the setting/prescription of cooler dialysate temperatures (not currently done). This would be 'Strategies Suggested for Implementation.'	e.g. prompts that already exist to prescribe a dialysate "Right now when we enter our orders whether it's for an inpatient or an outpatient, **in your prompts you have to put in a temperature**." (Physician#1)<br><br>"**You tell them, "Look, we're doing this because A, B and C and if you feel cold we'll give you an extra blanket.**" to have some mitigating factors for the subjective symptoms of being cold. Hopefully you can convince a fair amount of patients to actually agree to that." (Physician#1)
**15. Nature of Behaviour**<br>*What do you do and is that different from what you should do? (AP/JP)*		<u>Consider coding to this domain:</u> What participants usually do when prescribing or setting dialysate temperatures (Also code to Social Professional Role and Identity)<br><br>Descriptions of how participants are not currently 'individualizing' the dialysate temperatures.<br><br>Descriptions of how dialysate temperature is currently an automatic/default setting on machine.<br><br>Descriptions of how often cooler dialysate temperatures are prescribed or set in usual	"I have to say that at least not in my practice and I don't think in many practices **there's not a lot of people who actually look at the core patient's temperature to decide what they're going to set the dialysis temperature.** It's more, let's say patients having recurrent low blood pressure. You look at their prescription. They're at 36.5. You'll say let's drop it to 36. We're not going to look at what their core temperature is. We're just going to say, "Let's drop them by a .5 or a one degree towards the

		practice<br><br>Descriptions of when changes to dialysate temperatures usually occur	cooler side.”’ (Physician#1)

Topics often double-coded

Patient clinical factors: Environmental Context and Resources & Beliefs about Consequences

Accuracy of core body temperature reading: Environmental Context and Resources & Beliefs about Consequences

Unit standards/individualized cooler dialysate temperatures : Knowledge and Goals

Descriptions of what physicians and nurses normally do when prescribing and setting dialysate temperatures: Social Professional Role and Identity & Nature of Behaviour
