# Supplementary material for: Barriers and facilitators to healthcare professional behaviour change in clinical trials using the Theoretical Domains Framework: a case study of a trial of individualized temperature-reduced haemodialysis
Source: Trials. 2017 May 22;18:227. doi: 10.1186/s13063-017-1965-9 (PMC5440991; doi:10.1186/s13063-017-1965-9)
Supplement: Supplementary file 4 — Proposed strategies. (PDF 114 kb) [file 13063_2017_1965_MOESM4_ESM.pdf]

Additional File 4.

Proposed implementation strategies based on identified barriers and enablers to prescribing and setting individualized dialysate temperatures

**Proposed Implementation Strategies (with examples)**	**Corresponding belief statements (*TDF domains*)**
**1. Conduct structured unit assessments in all units** (Gather information about dialysis units, e.g., current unit policy, process to change unit policy, unit turnover rates, dialysis machines used, use of heated seats, what is routine educational resource on unit, who is respected local educator, increments of dialysate temperature change [i.e. 0.1 or 0.5 ºC])	- Individualized cooler dialysate temperatures will/may conflict with local policies *(Knowledge, Goals)*
	- Individualized cooler dialysate temperatures will not conflict with local policies *(Knowledge, Goals)*
	- Existing unit standards for dialysate temperatures vary between units *(Knowledge, Goals)*
	- Dialysis machine can be adjusted in 0.5 increments or less up to 35 (*Environmental Context & Resources*)
	- Variability in types of dialysis machines (*Environmental Context & Resources)*
	- Variability in how dialysate temperatures and prescriptions are recorded in EMR or paper charts (*Environmental Context & Resources)*
**2. Identify local champion, develop materials to train local unit champions and communication materials for local unit champions to use with unit staff** (e.g., slide deck for presenting study rationale and evidence, procedural steps for nurses to follow, examples of how the new procedure differs from existing routines, details of the trial timeline)	- Need better evidence of benefits of individualized dialysate temperature (*Knowledge*)
	- Need to demonstrate impetus for change (*Knowledge*)
	- The protocol must show convincing evidence for the study (*Knowledge*)
**3. Develop materials that nurses can provide to patients as required** (e.g., short explanation of study, impact on them, more personalized, long term benefits potential in the form of a patient letter)	- Patients feeling cold would influence me (*Beliefs about consequences*)
**4. Develop troubleshooting FAQ sheet** (e.g., Provide list of solutions to the identified potential barriers to be used as part of implementation strategy after	- Negative clinical management consequences (*Beliefs about consequences*)
	- Patients feeling cold would influence me (*Social Influences*)

randomization, and updated on on-going basis updated as required)	- Coping plans for patients who say they are cold (e.g., blankets) (*Behavioural Regulation*)
	- Climate in the winter or summer can impact the accuracy of core body temperature readings (*Environmental Context and Resources*)
	- Consumption of warm beverages or ice can influence core body temperature measurement (*Environmental Context and Resources*)
**5. Provide temperature-setting protocol**	- Easier to implement if protocol in place (*Beliefs about Capabilitie*s)
(e.g. develop two different temperature-setting protocols– for centres depending on whether they can change dialysate temperature by 0.1ºC. 0.5ºC, or both.)	- Need guidance on how to set temperature *(Knowledge)*
**6. Blanket dialysate prescription consistent with trial allocation at each centre**	- Individualized cooler dialysate temperatures will/may conflict with local policies *(Knowledge, Goals)*
**7. Ongoing engagement with local champion(s) at each centre** (identified in unit assessment, who can implement the MyTEMP trial at their respective units)	- Physicians influence views of individualized cooler dialysate temperatures (*Social Influences*)
	- Other nurses influence me or nurses influence each other (*Social Influences*)
	- It is important that staff buy-in (*Intention*)
**8. Train the Trainer** (Training sessions with unit champions about the protocol, how to set individualized temperatures, how to address identified barriers, and how to support staff in delivery of protocol)	- Physicians influence views of individualized cooler dialysate temperatures (*Social Influences*)
	- Other nurses influence me or nurses influence each other (*Social Influences*)
	- Education is necessary for implementation (Knowledge)
	- We need to know who is eligible or how to deal with various clinical factors (*Knowledge*)
	- We need guidelines or more information on procedures (*Knowledge*)
**9. Reminders** (add a visual cue to usual checks – e.g., sticky note, flag, or thermometer-shaped sticker. Provide a table with arithmetic for easy subtraction of 0.5 from measured temperature, to keep on or near dialysis machines)	- We would need reminders to set or prescribe individualized cooler dialysate temperatures (*Memory, Attention, Decision Processes*)
	- Setting individualized temperature requires mathematical thinking to subtract 0.5 from core temperature (*Memory, Attention, and Decision Processes*)

**10. Regular monthly monitoring**<br><br>(e.g., Local champion assess whether individualized cooler dialysate temperatures are being prescribed and set, assess any ongoing barriers to implementation, remind physicians and nurses, local champion to communicate with trial team; trial team to monitor monthly data on randomly selected patients)	- Auditing to ensure behaviour (*Behavioural Regulation*)
